# Lurasidone for Pediatric Bipolar Disorder: A Systematic Review

**DOI:** 10.3390/ph18070979

**Published:** 2025-06-30

**Authors:** Alexia Koukopoulos, Claudia Calderoni, Georgios D. Kotzalidis, Tommaso Callovini, Lorenzo Moccia, Silvia Montanari, Gianna Autullo, Alessio Simonetti, Mario Pinto, Giovanni Camardese, Gabriele Sani, Delfina Janiri

**Affiliations:** 1 Department of Life Science, Health and Health Professions, Link Campus University, 00165 Rome, Italy; a.koukopoulos@unilink.it (A.K.); g.camardese@unilink.it (G.C.); 2 Department of Psychiatry, Fondazione Policlinico Universitario Agostino Gemelli IRCCS, Largo Agostino Gemelli 8, 00168 Rome, Italy; claudia.calderoni1991@gmail.com (C.C.); giorgio.kotzalidis@gmail.com (G.D.K.); t.callovini@gmail.com (T.C.); lorenzomoccia27@gmail.com (L.M.); silvia.montanari@yahoo.com (S.M.); gianna.autullo@gmail.com (G.A.); or alessio.simonetti@bcm.edu (A.S.); mario.pinto@guest.policlinicogemelli.it (M.P.); gabriele.sani@unicatt.it (G.S.); 3 Department of Neuroscience, Section of Psychiatry, Università Cattolica del Sacro Cuore, Largo Francesco Vito 1, 00168 Rome, Italy; 4 Menninger Department of Psychiatry and Behavioral Sciences, Baylor College of Medicine, Houston, TX 77030, USA

**Keywords:** second-generation antipsychotics, lurasidone, bipolar disorder, mixed state, children, adolescents

## Abstract

**Background/Objectives**: Lurasidone ((3aR,4S,7R,7aS)-2-{(1R,2R)-2-[4-(1,2-benzisothiazol-3-yl)piperazin-1-ylmethyl]cyclohexylmethyl}hexahydro-4,7-methano-2H-isoindole-1,3-dione) is a second-generation antipsychotic approved for schizophrenia and mood disorders. Adolescents and children with bipolar disorder receive treatments that expose them to weight gain and metabolic syndrome. Lurasidone is relatively free from such side effects, so it may constitute a useful alternative for the treatment of these patients. We focused on the use of lurasidone in children and adolescents with bipolar disorder. **Methods**: On 11 June 2025, we used the following strategy on PubMed: lurasidone AND (“bipolar disorder” OR “bipolar depression” OR mania OR manic). We filtered for humans and ages 0–18 years and included case reports and clinical studies. Similar strategies adapted to each database were used to carry out our systematic review on CINAHL, PsycINFO/PsycARTICLES, Scopus, and the ClinicalTrials.gov register on the same date. We excluded reports without children/adolescent participants, those grouping adult participants with children/adolescents without providing data separately, reviews, and opinions/editorials with no data. Eligibility was determined through Delphi rounds; it was required that consensus was reached among all authors. We followed the PRISMA-2020 Statement. **Results**: Our search produced 38 results on PubMed on 11 June 2025. We included four case reports/series and five studies. One additional eligible study emerged from our Scopus inquiry, raising the number of eligible studies to six. One case series was moderately positive; one case report was neutral, another was positive, and one reported the induction of mania. The six longitudinal studies involved 16,735 participants and showed generally good efficacy. **Conclusions**: The use of lurasidone in adolescents/children with bipolar disorder obtains favorable results regarding the excitatory and depressive symptoms of bipolar disorder with no significant side effects.

## 1. Introduction

Bipolar disorder (BD) is a psychiatric disorder characterized by extreme mood fluctuations, including episodes of mania or hypomania and/or depression. Mood episodes tend to alternate in BD, but sometimes, they occur together; in these cases, we speak about mixed episodes, a specifier in the DSM-5-TR [1]. When a manic episode develops, the disorder is labeled type 1 (BD-I), but if there is alternation between major depressive episodes (MDEs) and hypomanic episodes with no occurrence of manic episodes in the patient’s history, this is considered to be the presence of bipolar disorder type 2 (BD-II). The lifetime prevalence of a definite BD diagnosis is about 2.2% in the general population [2], but BD-I tends to have a lower age at onset than BD-II, typically from 12 to 24 years [3]; hence, it is often of pediatric interest, as at least 25% of cases have their onset before age 20 [4].

In pediatric populations, BD tends to follow a more chronic and severe course compared to adult-onset forms, often leading to severe impairments in functioning, academic performance, and social relationships [5]. It is associated with alterations in emotional face recognition [6], emotional dysregulation [7], and a higher prevalence of childhood trauma [8,9], which also has distinctive brain correlates [10,11]. All this leads to an impellent need to treat pediatric BD, as this still constitutes an unmet need due to its high comorbidity rates in this patient population [12].

The management of pediatric bipolar disorder is challenging, especially when it comes to selecting medications that are both effective and have favorable side-effect and risk-to-benefit profiles, due to the potential for long-term impacts on a child’s development and overall health. In fact, BD is a life-long disorder, and its drug treatment is also likely to be life-long, but all the major pharmacological agents used in BD are likely to be associated with adverse events that limit the clinical effectiveness of these drugs. This is particularly important in a child/adolescent, whose brain is still maturing. Traditionally, bipolar disorder has been treated with mood stabilizers and first-generation antipsychotics, which are often associated with significant adverse effects, including weight gain, metabolic syndrome, and extrapyramidal symptoms [13].

**Figure 1:**
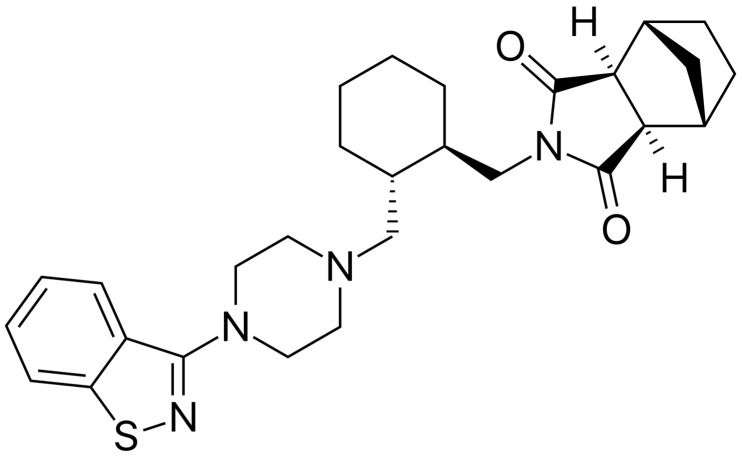
The chemical structure of lurasidone.

Lurasidone ((3aR,4S,7R,7aS)-2-{(1R,2R)-2-[4-(1,2-benzisothiazol-3-yl)piperazin-1-ylmethyl]cyclohexylmethyl}hexahydro-4,7-methano-2H-isoindole-1,3-dione) (Figure 1) is a second-generation antipsychotic with high-affinity antagonism at D_2_, 5-HT_2A_, and 5-HT_7_ receptors and partial agonism at 5-HT_1A_, which, differently from other atypical antipsychotics, is more potent at the D_2_/D_3_ group of dopamine receptors than at serotonin 5-HT_2A_ receptors [14,15], which has demonstrated antidepressant efficacy in adult bipolar I depression, both as a monotherapy [16] and as an adjunct to lithium or valproate [17], without the pronounced metabolic side effects which are typical of many atypical antipsychotic drugs [18]. This second-generation antipsychotic also appears to be associated with a relatively low risk of weight gain, making it a potential candidate for the treatment of children and adolescents with BD [19]. Many neuroleptics and antipsychotics display a piperazine structure, but not all of them are alike in their neurochemical profiles [20]. The favorable cardiometabolic profile of lurasidone [18,19] makes this second-generation antipsychotic drug a serious candidate for use in pediatric BD. The drug received approval from the US Food and Drug Administration (FDA) in 2020 for schizophrenia and bipolar disorder and in Europe from the European Medicines Agency (EMA) in 2020, to treat children aged 13 years or more. Lurasidone is not recommended for children less than 10 years of age; according to the EMA, the starting dose should be 18.5 mg/day with a recommended maximum dose of 148 mg/day. It should be prescribed by an expert in pediatric psychiatry and must be taken with at least 350 kcal food.

The safety and efficacy of lurasidone in younger populations, particularly in those aged up to 18 years, remain of interest, but the existing literature is still limited. Early studies on lurasidone’s pharmacokinetics and tolerability in children and adolescents with psychiatric disorders have shown promising results [21,22], indicating that the medication may be well tolerated with a relatively favorable side-effect profile [21]. Furthermore, studies specifically investigating lurasidone in pediatric bipolar depression have suggested potential therapeutic benefits as a monotherapy [20]. Additionally, as demonstrated in adult studies, the use of lurasidone with mood stabilizers, like lithium or valproate [16], suggests potential utility in pediatric populations. These findings indicate that lurasidone may help to improve depressive symptoms in pediatric patients with BD, providing an effective alternative to traditional therapies that are linked to a higher risk of metabolic side effects.

A recent systematic review [23] compared the efficacy and safety of second-generation antipsychotics, including lurasidone, with other treatment options for pediatric bipolar disorder. The review found that lurasidone may be comparable to, or even superior to, other antipsychotics in terms of safety, particularly due to its significantly lower risks of metabolic side effects and extrapyramidal symptoms.

Bipolar disorder in children and adolescents represents a significant challenge for clinicians, as it requires a more careful balance between effective symptom management and minimizing the potential side effects.

According to the guidelines set forth by Yatham et al. [24], traditional treatments, which include mood stabilizers like lithium and anticonvulsants like valproate, as well as first-generation antipsychotics, are effective in managing symptoms but frequently come with side effects, including weight gain, sedation, and metabolic issues. These side effects are particularly concerning in the pediatric population, where they may impact growth, academic performance, and overall well-being. As a result, there is a growing interest in exploring alternative pharmacological treatments that are both effective and have a more favorable side-effect profile.

For children and adolescents, the use of lurasidone has been studied primarily in the context of bipolar I depression, with results suggesting that it is well tolerated and can significantly reduce depressive symptoms.

As children and adolescents are still in developmental stages, the long-term impact of medications like lurasidone on growth, cognitive function, and overall quality of life is not fully understood. Furthermore, while some studies have highlighted its safety profile, others have reported instances of manic switch or insufficient efficacy in certain patients, necessitating continued vigilance and research to optimize treatment protocols [25,26].

Despite these promising findings, the use of lurasidone in pediatric populations is not without concerns. There have been occasional reports of adverse effects, including manic switch, sedation, and gastrointestinal disturbances [27]. These side effects, while generally mild and transitory, highlight the need for more large-scale studies to establish clear guidelines for the optimal use of lurasidone in treating pediatric bipolar disorder.

Given the limited evidence on the use of lurasidone in pediatric bipolar disorder, the objective of this systematic review is to comprehensively assess the current evidence on the efficacy and safety of lurasidone in the treatment of pediatric bipolar disorder. Specifically, this review aims to evaluate lurasidone’s therapeutic potential, side-effect profile, and tolerability in children and adolescents. Case reports, clinical trials, and longitudinal studies are examined to assess both the therapeutic potential and safety profile of lurasidone in managing bipolar disorder in pediatric patients.

## 2. Methods

*Search strategy and information sources:* To investigate the use of lurasidone in pediatric BD, we searched the PubMed database on 9 May 2025, using the following search strategy: lurasidone AND (“bipolar disorder” OR “bipolar depression” OR mania OR manic). The search produced 268 results, but limiting to ages 0–18 we obtained 38 records, shown in Supplementary Table S1. We further searched the CINAHL, PsycINFO/PsycARTICLES, and Scopus databases and the ClinicalTrials.gov register using appropriate search methods for each database. Eligibility was determined by all participating authors, who discussed it through Delphi rounds. No more than two were required to obtain consensus on eligibility. We applied the PRISMA 2020 Statement in conducting our review [28].

*Criteria for eligibility and selection of studies:* Studies or case reports dealing with the administration of lurasidone to patients of pediatric age (0–18 years old, i.e., children and adolescents) with BD were eligible.

**Figure 2:**
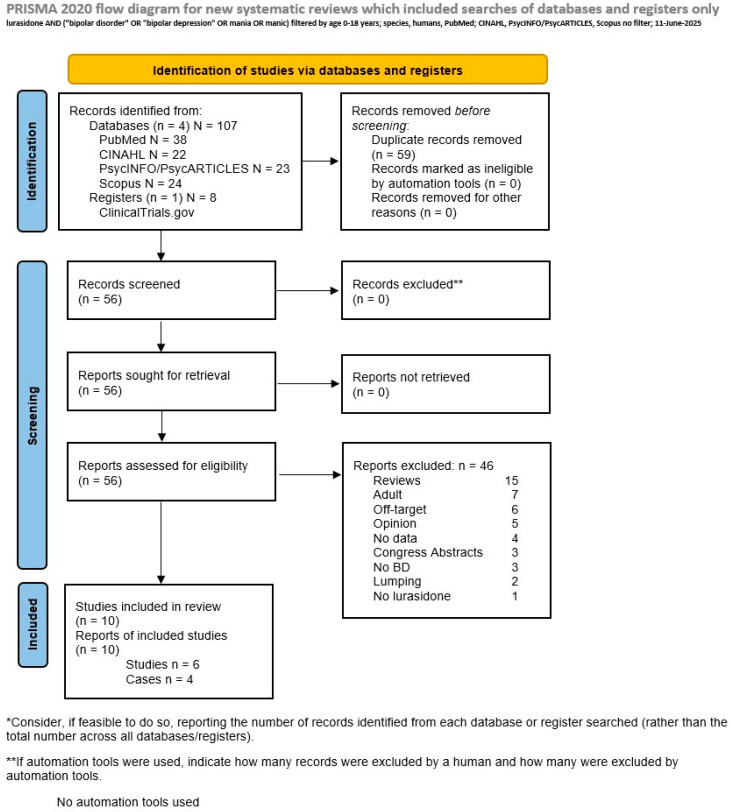
Flowchart of our search strategy according to the PRISMA Statement [28]. Source: Page MJ, et al. *BMJ* 2021;372:n71. doi: 10.1136/bmj.n71. This work is licensed under CC BY 4.0. To view a copy of this license, visit https://creativecommons.org/licenses/by/4.0/ (accessed on 11 June 2025).

*Exclusion criteria:* We excluded reviews (labeled Review; however, we retrieved them and hand-searched for eligible papers possibly missed by our research strategy), editorials or letters to the editor expressing the author’s opinions but not providing data, labeled Opinion, studies that were included in the search but dealt with adult patients, termed Adult, studies which, despite including children/adolescents, grouped their results with those of adults (termed Lumping), studies not using lurasidone (No lurasidone), studies not including BD patients (No BD), studies without data (No data), and abstracts presented at congresses and meetings (Congress Abstract). Articles not specifically targeting the efficacy or safety of lurasidone in pediatric BD were labeled as Off-target, which comprised the unfocused and unrelated to the subject matter categories. We also would have excluded retired or retracted papers and those that present data on the same samples, termed as Overlapping, keeping only those of the best quality, or studies presenting the same data or corrections to an existing article, termed Duplicates; however, no such categories emerged for PubMed. Duplicates among the investigated databases/registers were eliminated (Supplementary Table S1). We did not restrict our searches to a particular time frame (all databases were searched since their inception) and imposed no language restrictions. We show the selection process in Figure 2, where we present the PRISMA flowchart of our search.

*Review registration on a platform:* We registered our review on the Open Science Framework (OSF) platform, with the following registration identifier: DOI: 10.17605/OSF.IO/84CT6 (accessed on 11 June 2025).

*Quality and risk-of-bias assessment:* We examined the quality of the case reports with the JBI Critical Appraisal Checklist for Case Reports [29,30] and the risk of bias of the included studies with the Cochrane risk-of-bias (RoB-2) tool [31]. The results are shown in Supplementary Table S3.

## 3. Results

*The literature search and screening and data extraction:* Our above PubMed search on 9 May 2025 produced thirty-eight results, of which nine were eligible, four case reports and five clinical studies, while with the addition of other databases, one more study was found to be eligible, bringing the grand total to six. Twenty-nine articles were excluded, specifically the following: thirteen Reviews, five Off-target, five Opinions, five Adult, and two Lumping. The CINAHL search yielded 22 articles, and the search on the American Psychological Association databases PsycINFO and PsycARTICLES produced 23 articles, using the “lurasidone AND bipolar disorder AND (pediatric or child or children or infant or adolescent)” strategy for both CINAHL and PsycINFO/PsycARTICLES, while on Scopus, the “lurasidone [title] AND bipolar disorder [title, abstract, keywords] AND (children OR adolescents) [title, abstract, keywords]” search produced 24 articles. Only the last search produced one more eligible article, for a grand total of ten studies, four cases and six controlled trials. The results of our search are shown in Figure 2.

*Case reports/series:* Regarding the four case reports [27,32,33,34], the first three referred to one patient each, and the fourth one referred to six patients (Table 1). Of the nine patients described here, one was from Singapore and of Chinese ethnicity [27], while the others were presumably white, but this was not specified in the articles. One of these reports was conducted in the US [32], another presumably in the UK [33], one in Singapore [27], and the multi-case one in Australia [34]. Five of the patients were female, while three were male, and one was a transgender male [34]. The Chinese BD-I patient received lurasidone to treat a depressive relapse after receiving stabilizers with risperidone, olanzapine, and electroconvulsive therapy (ECT), but he developed a manic episode and discontinued. The other cases were more favorable to lurasidone regarding both manic and depressive symptoms.

*Clinical studies:* The six clinical studies involved 16,735 patients overall (Table 2). One, the most numerous, was a pharmacoeconomic study conducted on a US database, which compared hospitalizations after atypical antipsychotic prescriptions [35], while two were conducted by the same group [19,26] and were international and coordinated by the Cincinnati group; they were conducted in the US, Europe, Mexico, Colombia, South Korea, and the Philippines. The first was a 6-week study; the second a 104-week extension. The other two US studies [25,36] were post hoc analyses of the DelBello et al. study [19]. Despite being centered on the same patient population, we used these studies because they focused on different outcomes. A further Chinese study involved a comparison of lurasidone vs. quetiapine and focused primarily on cognitive outcomes but also measured depression and found the two drugs to perform similarly across all outcomes [37].

Examining the quality of the eligible case reports, we found them all to be high-quality studies according to the measures we used [29,30], while the studies included were mostly free of bias according to the Cochrane RoB-2 tool [31] (Supplementary Table S3). Overall, the quality of the eligible studies and case reports was high, and they were bias-free, despite many of the former being industry-sponsored.

While the clinical and pharmacoeconomic studies obtained a favorable profile for the use of lurasidone in pediatric BD, the case reports suggest more caution, as one of them was unenthusiastic and another even reported the induction of mania in a patient of Chinese origin when trying to counter bipolar depression.

## 4. Discussion

In this review of lurasidone in pediatric BD, we obtained favorable results in randomized controlled trials (RCTs) and a pharmacoeconomic study focusing on hospitalization in comparison with other atypical antipsychotics. The results stemming from case reports also favor the use of lurasidone, although with some inconsistencies. One study found manic excitement in a Chinese patient after an attempt to counter bipolar depression after five ECT sessions and various unsuccessful antipsychotic trials involving olanzapine and risperidone [25]. This previously unreported effect related to lurasidone use should be further investigated as to its ethnic/racial implications.

As mentioned, bipolar disorder in children and adolescents comes with a significant burden, including early onset, mixed or rapid cycling patterns, and common co-occurring conditions such as anxiety, ADHD, and neurodevelopmental disorders [3], all of which lead to severe functional impairments and an increased risk of suicide [38,39]. The main challenge in treating this age group is achieving remission from depressive symptoms while avoiding a manic switch or side effects [22]. Lurasidone, with its potent antagonism at D_2_ and 5-HT_2_A receptors, along with 5-HT_7_ antagonism and minimal affinity for H_1_ and M_1_ receptors, offers both strong antidepressant efficacy and a more favorable metabolic profile compared to many second-generation antipsychotics [40,41,42,43].

The results of the randomized controlled trials [19,26] demonstrate that lurasidone is efficacious in reducing depressive symptoms in children and adolescents with BD, even in those with mixed features. The pharmacoeconomic study [35] is witness to the effectiveness of the drug in a real-world setting. The six-week RCT by DelBello et al. [19], involving 347 youths (aged 10–17 years), showed that flexible doses of lurasidone (20–80 mg/day) resulted in a significant reduction in depressive symptoms compared to the placebo, with benefits evident by the second week of treatment. Lurasidone could already separate from the placebo groups by week 2 and maintained the advantage throughout the study. Additionally, a subsequent network analysis by Singh et al. [37] identified sleep disturbance and irritability as symptoms linking depressive and manic symptom clusters. Targeting these symptoms with lurasidone led to superior outcomes, particularly in patients with sleep disturbance. These authors also observed that lurasidone improved both depressive and manic symptom clusters, as assessed through the Children’s Depression Rating Scale—Revised (CDRS-R) and the Young Mania Rating Scale, respectively, and global functioning assessed through the Children’s Global Assessment Scale (CGAS). Furthermore, they noted changes in the symptomatic remission (CDRS-R ≤ 28) and functional remission (CGAS ≥ 71) rates over time, highlighting the medication’s effectiveness in addressing both depressive symptoms and functional impairments. Similar response/remission rates were found for Chinese patients who took lurasidone *vs*. quetiapine for 8 weeks; both drugs were similarly efficacious [36].

Additionally, the 104-week open-label extension of this study [26] found that 55% of participants stayed on lurasidone for two years, indicating that patients were relatively treatment-adherent/compliant. It must be stressed that patients with BD show the least medication compliance among psychiatric patients, with no more than half of patients adhering to their prescription [44]. These patients have maintained improvements in both depressive symptoms (CDRS-R) and global functioning (CGAS) with minimal weight gain. The participants also showed stable metabolic measures, including lipids, glycemic levels, and prolactin, suggesting that lurasidone has a favorable metabolic profile. This stability contrasts with the metabolic side effects often associated with the long-term use of other antipsychotics, like olanzapine or risperidone [45], underlining lurasidone’s value, particularly for those who are at high cardiometabolic risk [26].

Kadakia et al. [35] analyzed 16,201 pediatric patients with bipolar disorder and found that lurasidone was associated with a significantly lower risk of psychiatric hospitalization compared to aripiprazole and olanzapine, although it did not outperform quetiapine or risperidone. The pharmacoeconomic advantage found for lurasidone in terms of reduced hospitalization savings is paralleled by the favorable data of other pharmacoeconomic studies on lurasidone in adult patients [46,47].

In Singh et al.’s [25] post hoc analysis, DelBello et al.’s [19] double-blind, placebo-controlled RCT was reanalyzed, involving 347 adolescents aged 10–17 years with DSM-5 bipolar I depression. Lurasidone flexibly dosed at between 20 and 80 mg/day significantly improved depressive symptoms, as measured by the CDRS-R at week 6. Improvements were evident regardless of the presence of mixed features, and additionally, global functioning measured by the CGAS improved, reinforcing the role of lurasidone not only in symptom reduction but in increasing day functioning. The safety profile was comparable to that of the placebo, with no significant metabolic or prolactin-related adverse effects, lining up with previous pediatric trials. These improvements resemble those obtained by Kato et al. [48], who conducted a large, multicenter RCT with 525 adult patients (aged 18–74 years) with bipolar I depression. They also confirmed lurasidone’s antidepressant efficacy and favorable metabolic profile in adult bipolar depression, with no added benefits at higher doses. These findings support the potential application of lurasidone in younger populations.

Case reports and case series can help show how lurasidone may hold particular value in complex, treatment-resistant adolescent psychiatric cases, where polypharmacy risks and adverse events limit conventional antipsychotic use. In one of these [32], a 14-year-old girl with early-onset psychosis and a complex psychiatric history, which included anorexia nervosa, experienced significant improvement in psychotic symptoms, mostly auditory hallucinations, paranoia, and functional impairment, after lurasidone was titrated up to 148 mg/day. Importantly, lurasidone was well tolerated, with minimal sedation, no extrapyramidal symptoms, and stable weight over 8 weeks, in contrast to prior adverse effects encountered with olanzapine, risperidone, and aripiprazole. This case emphasizes lurasidone’s potential utility in youth with psychosis who are sensitive to weight gain, sedation, and prolactin-related adverse effects. Similarly, another case report [33] brought to attention a case of a 16-year-old boy with bipolar I disorder, where persistent sleep disturbance and associated irritability persisted despite mood stabilization on valproate and lurasidone. The addition of suvorexant, a dual orexin receptor antagonist, improved sleep duration and quality, with further benefits regarding irritability, aggression, and academic performance which were sustained for 7 months without adverse events. While lurasidone alone did not resolve the insomnia, its ability to maintain mood stability without significant side effects allowed for supplementary off-label interventions like suvorexant, an option typically limited by tolerability concerns in polypharmacy in adolescent bipolar disorder.

In a case series involving six patients between the ages of 14 and 17 years from the Community Child and Adolescent Mental Health Services (CAMHS), who presented a wide range of complex psychiatric conditions, including autism-related irritability, personality disorder traits, post-traumatic stress disorder (PTSD), and genetic syndromes, three patients showed significant clinical improvement, two showed marginal progress, and none experienced weight gain, metabolic abnormalities, or prolactin elevation [34]. In particular, three patients with severe mood dysregulation, despite prior use of risperidone, quetiapine, and mood stabilizers, experienced improvements in school attendance and social interaction after switching to lurasidone. In particular, the low weight gain potential of lurasidone compared to other second-generation antipsychotic agents could be due to its lower 5-HT_2C_ blocking activity compared to its 5-HT_2A_ serotonin receptor antagonist activity [49,50]. In fact, drugs endowed with agonist 5-HT2C activity counteract weight gain [51]. These positive outcomes were obtained without sedation or cardiometabolic side effects, underlining the potential of lurasidone in effectively managing treatment-resistant, complex psychiatric conditions within CAMHS settings.

One important finding of our review is the low akathisia rate seen in pediatric BD populations [19,26,32]. Akathisia is a frequent occurrence in adult patients with schizophrenia treated with lurasidone (about 15%) [52] and, generally, does not differ in occurrence with other drugs between schizophrenia and BD samples [53]. It is difficult to say whether its occurrence rates differ between adults and adolescents, as agitation and hyperactivity may be confounders for akathisia in pediatric populations [54].

It is important to monitor the treatment of emergent mania when using lurasidone. A case reported by Nair et al. [27] described a 17-year-old Chinese boy in Singapore who, just five days after starting lurasidone 20 mg in combination with valproate and olanzapine, developed a severe manic episode with psychotic symptoms. Despite treatment with anti-manic medications, his symptoms persisted and required ECT with stabilization occurring only after lurasidone was discontinued [27]. Although clinical trials in pediatric populations have shown similar rates of mania switch between lurasidone and the placebo [55], certain pharmacodynamic factors beyond liver CYP2D6 metabolism, which is not affected by lurasidone [56], such as individual baseline dopaminergic tone or single-nucleotide polymorphisms, and the use of concurrent antidepressants may increase vulnerability to this side effect [57], particularly when doses are escalated quickly or in cases of comorbidity or rapid cycling illness [58].

Overall, lurasidone demonstrates significant antidepressant efficacy in pediatric bipolar depression, with a good metabolic and tolerability profile. Its long-term safety, coupled with real-world evidence supporting its use in complex clinical cases, reinforces its position as a valuable option in this population.

**Limitations**: The major limitation of this review is the dearth of studies focusing on lurasidone in pediatric BD. The limited sample sizes of the clinical trials, despite the inclusion of a large sampled database, further add to the limitations. Two of the included studies were *post hoc* and conducted on the same sample. We avoided counting these cases among the total figures of patients involved in our eligible studies. These facts rendered a meta-analysis unfeasible. Another limitation could be our restriction to only four databases, but it is unlikely that other databases could add eligible studies. Despite these limitations, this is the first systematic review of the subject and should help clinicians seeking to find safe and effective means to treat their child and adolescent patients with BD. The strengths of this study include the investigation of four databases and one register, the eligible studies’ high quality and low risk of bias, and its registration on a platform.

***Future research perspectives***: Summarizing our research data, we may state that future research should focus on identifying the body weight reduction potential of lurasidone and its neurochemical underpinnings, on the real-world effectiveness of this molecule in pediatric populations with depression and/or BD, and on verifying the real occurrence of extrapyramidal symptoms and akathisia in these populations. In particular, the latter poses differential diagnosis problems that will have to be resolved through clinical testing. The issue of whether akathisia is observed less in children/adolescents vs. adults or in BD vs. schizophrenia requires further attention and experimentation.

## 5. Conclusions

In conclusion, lurasidone has emerged as a promising pharmacological treatment for pediatric bipolar depression, offering both rapid and sustained antidepressant effects across randomized, placebo-controlled trials, as well as in real-world settings. However, even though the overall risk of adverse events is low, it is still important to monitor and report any side effects, especially manic switches. The current evidence supports lurasidone as a promising option for pediatric bipolar depression, combining efficacy with a favorable safety profile, but whether it can be considered among first-line interventions will be determined by further studies. This confirms its value as a key component in appropriate treatment plans. When integrated into an approach that combines evidence-based psychotherapy, family-focused interventions, and targeted sleep management, lurasidone provides a subtle yet effective solution to the challenge of managing bipolar disorder in children and adolescents.

## Figures and Tables

**Table 1:** Summary of clinical case reports/series of patients of pediatric age (childhood and adolescence) receiving lurasidone in chronological order.

Author(s)/Year	Patient(s)	Treatment	Outcomes
Channing et al., 2018 [32]	14 y.o. female; early-onset psychosis (schizophrenia criteria), past anorexia nervosa, persistent delusions, multimodal hallucinations (auditory, visual, tactile, olfactory), paranoia, social withdrawal, and low mood.	Olanzapine (sedation, weight gain), risperidone (extrapyramidal symptoms, elevated prolactin, nosebleeds), and aripiprazole (partial response, sedation, restlessness, weight gain). Lurasidone was started at 18.5 mg/day, titrated by 18.5 mg every 3–4 days up to 148 mg/day over 6 weeks, with procyclidine co-administered initially.	Symptom improvement after one week at 148 mg/day, reduced voices, diminished paranoia, and improved function. Maintained gains at discharge. Minimal sedation, no akathisia, and no extrapyramidal symptoms. Weight stable (0.5 kg increase over 8 weeks), with weight initially dropping during illness-related poor intake.
Prieto et al., 2019 [33]	16 y.o. male; bipolar I disorder. Persistent insomnia despite mood stabilization (3–4 h/night), irritability, low frustration tolerance, and aggression at home/school.	Valproate 1250 mg/day (therapeutic), lurasidone 140 mg/day (stable mood), and prior failed trials of melatonin 10 mg, trazodone 50 mg, quetiapine 800 mg, and clonazepam 0.5–1 mg. Suvorexant 10 mg nightly, off-label use with informed consent.	Sleep improved to 6–7 h/night, better sleep quality, less daytime irritability and aggression, and improved school performance. Benefits sustained over 7 months without tolerance or dosage increase. Well tolerated, no daytime sedation, and no adverse effects reported over 7 months.
Nair et al., 2021 [27]	17 y.o. male (Chinese); bipolar I disorder, prior misdiagnoses of adjustment disorder and MDD. History of psychotic mania with grandiose delusions, hallucinations, hypersexuality, irritability, and poor sleep.	Fluoxetine (20 mg, induced mania), sodium valproate (up to 1000 mg), olanzapine (up to 15 mg, limited effect), haloperidol, risperidone (ineffective), and 5 ECT sessions (initial remission), followed by depressive relapse. Lurasidone started at 10 mg, increased to 20 mg/day over 3 days.	Developed severe manic episode with psychotic features within 5 days of starting lurasidone, despite concurrent valproate and olanzapine. Lurasidone discontinued; 12 further ECT sessions administered. Stabilized subsequently on lithium (1000 mg) + quetiapine (800 mg). Probable treatment-emergent mania linked to lurasidone. No documented extrapyramidal symptoms or metabolic adverse effects during brief exposure.
Mole et al., 2022 [34]	Case 1: 16 y.o. male; epilepsy, learning disability, ADHD, anxiety, autism, irritability, and anger dyscontrol. Case 2: 16 y.o. female; depression, emerging EUPD traits, poor sleep, recurrent overdoses, and chronic self-harm. Case 3: 14 y.o. trans male; gender dysphoria, depression, anxiety, substance use history, emerging EUPD, and self-harm. Case 4: 17 y.o. female; EUPD, moderate depression, chronic suicidality, anger outbursts, and prior hospitalizations. Case 5: 17 y.o. female; PTSD, EUPD, anxiety, emotional dysregulation, obesity, chronic suicidality, and self-harm. Case 6: 17 y.o. female; CHARGE syndrome, cerebral palsy, sleep apnea, learning difficulties, depressive symptoms, aggression, and self-harm.	Case 1: Risperidone (10 kg weight gain), fluoxetine, topiramate, melatonin, group therapy, and behavioral support. Lurasidone started at 20 mg, increased to 60 mg/day. Case 2: Sertraline, quetiapine, melatonin, and psychological therapy. Lurasidone 40 mg/day added to existing drugs. Case 3: Fluoxetine, sertraline, desvenlafaxine, and olanzapine (10 kg weight gain, no benefit). Switched to lurasidone (dose not specified). Case 4: Lamotrigine, risperidone (galactorrhea), paliperidone (galactorrhea), and quetiapine (weight gain). Lurasidone 40 mg/day, monotherapy after 6 months. Case 5: Sertraline, quetiapine (appetite/weight gain), and psychotherapy. Switched to lurasidone 40 mg/day. Case 6: Risperidone (hyperprolactinemia, galactorrhea) and clonidine. Switched to lurasidone, started at 40 mg/day, increased to 60 mg/day.	Case 1: Marked improvement in aggression and irritability. Better daily functioning and school engagement. Rebound insomnia, managed with sleep hygiene and short-term promethazine. There were no other side effects. Case 2: Adherence good. Equivocal clinical response. No significant change noted at 4 weeks. No adverse effects reported. Case 3: Reduction in irritability but no effect on hallucinations or self-harm. Discontinued by patient after 4 months. No adverse effects. Stable weight. Case 4: Marked improvement in mood, emotional lability, and anger. Stable for 18 months on monotherapy. No galactorrhea, sedation, or weight gain. Well tolerated. Case 5: Subjective mood stabilization and anxiety reduction. Facilitated engagement in intensive day program. No weight gain, metabolic abnormalities, or side effects. Rebound insomnia is managed with mirtazapine 7.5 mg. Case 6: Improved mood instability and depressive symptoms, but agitation persisted despite dose increase. No side effects reported up to 2 months on higher dose.

*Abbreviations:* BD, bipolar disorder; BID, bis in die, twice a day; FU, follow-up; ECT, electroconvulsive therapy; EUPD, Emotionally Unstable Personality Disorder; GAD, generalized anxiety disorder; ICU, intensive care unit; LC, liquid chromatography; pt(s), patient(s); SCZ, schizophrenia; SCZF, schizophreniform disorder; PL(s), plasma level(s); wk(s), week(s); y.o., years old; yr(s), year(s); ×, for, per; →, then, followed, after, subsequently; ↑, increased, augmented, rise, potentiated, stronger; ↓, reduced, reduction, diminished, lower, weaker; ↔, unchanged, no significant variation; ♀, female, girl, woman; ♂, male, boy, man.

**Table 2:** Summary of clinical studies on lurasidone during childhood and adolescence in chronological order.

Author(s)/Year	Population	Design	Treatment	Outcomes	Conclusions
DelBello et al., 2017 [19]	347 BD patients (lurasidone: 175; placebo: 172), ages 10–17, with bipolar depression	RCT, double-blind, placebo-controlled RCT	Lurasidone (10–20 mg/day for 10–14-year-olds; 20–40 mg/day for 15–17-yr-olds) × 6 wks.	Significant improvement in depressive symptoms on the CDRS-R and CGI-BP-S. ↑ response rates on CDRS-R for lurasidone.	Lurasidone has shown significant efficacy in ↓ symptoms of bipolar depression in children and adolescents compared to placebo. Safety profile acceptable; common side effects of nausea and somnolence. Akathisia less than with placebo. No cognitive or metabolic concerns.
Singh et al., 2020 [25]	347 patients (ages 10–17) with DSM-5 bipolar I depression	*Post hoc* of an RCT, double-blind, placebo-controlled study (DelBello et al., 2017 [19])	Lurasidone monotherapy, flexibly dosed 20–80 mg/day vs. placebo. Initial dose 20 mg/die. No adjunct mood stabilizers/antidepressants allowed.	Change in CDRS-R total score at wk 6. Change in CGI-BP-S, YMRS, PARS, CGAS, and PQ-LES-Q.	Lurasidone was effective in ↓ depressive symptoms in adolescents, regardless of the presence of mixed features. Safety profile comparable to placebo.
DelBello et al., 2021 [26]	306 participants (aged 10–17 years) with bipolar I depression	Double-blind RCT, placebo-controlled lurasidone trial, extension trial to 104 wks	Flexible-dose lurasidone 20–80 mg/day × 104 wks, starting at 40 mg/day for 1 wk, adjusted weekly for efficacy/tolerability. Mean dose: 52.1 mg/day. Concomitant benzodiazepines (16.7%) and stimulants (15.4%).	Long-term safety and tolerability (adverse events, metabolic parameters, extrapyramidal symptoms). Effectiveness on depressive symptoms (CDRS-R) and global functioning over 104 weeks.	Lurasidone was safe, well tolerated, and effective over 2 years in youth with bipolar depression. Low discontinuation, minimal metabolic or prolactin effects, low akathisia and extrapyramidal symptom rates, and sustained improvement in depressive symptoms and functioning.
Kadakia et al., 2021 [35]	16,201 pediatric patients (≤17 years) with DSM-5 bipolar disorder	Retrospective pharmacoeconomic cohort study	Oral atypical antipsychotics: lurasidone, aripiprazole, olanzapine, quetiapine, and risperidone.	All-cause hospitalization (any inpatient stay). Psychiatric hospitalization (any hospitalization with a psychiatric diagnosis, including bipolar disorder, depression, anxiety, ADHD, adjustment disorder, disruptive behavior, and other mental health conditions).	Lurasidone associated with a significantly ↓ risk of all-cause and psychiatric hospitalizations compared to aripiprazole and olanzapine, but not quetiapine or risperidone, suggesting a favorable real-world hospitalization profile for pediatric bipolar care.
Diao et al., 2022 [36]	61 10–17-yr-olds (lurasidone, n = 29 [6 ♂, 23 ♀, mean age, 14]; quetiapine, n = 32 [10 ♂, 22 ♀, mean age, 15]), age *p* < 0.035	Prospective longitudinal double-blind RCT	Lurasidone (20 mg/day gradually titrated to 60 mg/day in 1 wk) vs. quetiapine (100 mg/day gradually titrated to 300 mg/day in 1 wk) × 8 wks.	Primary outcome: cognitive performance (THINC Integrated Tool); secondary outcomes: response/remission rates in depressive symptoms (HAM-D ≥ 50%↓ score from BL and <final score ≤ 7, respectively), metabolic measures.	At the 8-wk FU, the two drugs did not differ in any of the measures assessed. There was a high attrition rate, with only 31 completers.
Singh et al., 2023 [37]	347 patients (ages 10–17) with DSM-5 bipolar I depression	Post hoc of an RCT, double-blind, placebo-controlled study (DelBello et al., 2017 [18])	Lurasidone monotherapy, doses of between 20 and 80 mg/day, or placebo, once daily for 6 weeks. All completers entered a 2-year open-label lurasidone extension.	Changes in depressive and manic symptom clusters (CDRS-R, YMRS) and global functioning (CGAS). Changes in rates of symptomatic remission (CDRS-R ≤ 28) and functional remission (CGAS ≥ 71) over time. Focus on “bridge” symptoms (decreased need for sleep, irritability) influencing outcomes.	Lurasidone improved sleep disturbance and irritability and overall depression, particularly in patients with sleep disturbance.

*Abbreviations:* BD, bipolar disorder; BID, bis in die, twice a day; CDRS-R, Children’s Depression Rating Scale—Revised; CGAS, Children’s Global Assessment Scale; CGI-BP-S, Clinical Global Impressions—Bipolar-Specific; FU, follow-up; GAD, generalized anxiety disorder; HAM-D, 17-item Hamilton Depression Rating Scale; ICU, intensive care unit; LC, liquid chromatography; pt(s), patient(s); SCZ, schizophrenia; SCZF, schizophreniform disorder; PARS, Pediatric Anxiety Rating Scale; PL(s), plasma level(s); PQ-LES-Q, Pediatric Quality of Life Enjoyment and Satisfaction Questionnaire; RCT, randomized controlled trial; wk(s), week(s); YMRS, Young Mania Rating Scale; yr(s), year(s); ×, for, per; →, then, followed, after, subsequently; ↑, increased, augmented, rise, potentiated, stronger; ↓, reduced, reducing, reduction, diminished, lower, weaker; ↔, unchanged, no significant variation; ♀, female, girl, woman; ♂, male, boy, man.

## Data Availability

All data are in the manuscript/Supplemental Materials and in the cited articles.

## Supplementary Materials

The following supporting information can be downloaded at https://www.mdpi.com/article/10.3390/ph18070979/s1: Supplementary Table S1. Records obtained in our PubMed, CINAHL, PsycINFO/PsycARTICLES, Scopus, and ClinicalTrials.gov searches and decisions for eligibility or exclusion, with reasons for exclusion (3rd column). Supplementary Table S2. PRISMA 2020 Checklist. Supplementary Table S3. Quality assessment of included studies.

## References

[1] American Psychiatric Association (2022). Diagnostic and Statistical Manual of Mental Disorders, Fifth Edition, Text-Revision (DSM-5-TR).

[2] Merikangas K.R., Akiskal H.S., Angst J., Greenberg P.E., Hirschfeld R.M., Petukhova M., Kessler R.C. (2007). Lifetime and 12-month prevalence of bipolar spectrum disorder in the National Comorbidity Survey replication. Arch. Gen. Psychiatry.

[3] Baldessarini R.J., Tondo L., Vazquez G.H., Undurraga J., Bolzani L., Yildiz A., Khalsa H.M., Lai M., Lepri B., Lolich M. (2012). Age at onset versus family history and clinical outcomes in 1665 international bipolar-I disorder patients. World Psychiatry.

[4] Faedda G.L., Baldessarini R.J., Suppes T., Tondo L., Becker I., Lipschitz D.S. (1995). Pediatric-onset bipolar disorder: A neglected clinical and public health problem. Harv. Rev. Psychiatry.

[5] Birmaher B. (2013). Bipolar disorder in children and adolescents. Child Adolesc. Ment. Health.

[6] Simonetti A., Lijffijt M., Kahlon R.S., Gandy K., Arvind R.P., Amin P., Arciniegas D.B., Swann A.C., Soares J.C., Saxena K. (2019). Early and late cortical reactivity to passively viewed emotional faces in pediatric bipolar disorder. J. Affect. Disord..

[7] Janiri D., Moccia L., Conte E., Palumbo L., Chieffo D.P.R., Fredda G., Menichincheri R.M., Balbi A., Kotzalidis G.D., Sani G. (2021). Emotional dysregulation, temperament and lifetime suicidal ideation among youths with mood disorders. J. Pers. Med..

[8] Janiri D., Moccia L., Montanari S., Simonetti A., Conte E., Chieffo D., Monti L., Kotzalidis G.D., Janiri L., Sani G. (2023). Primary emotional systems, childhood trauma, and suicidal ideation in youths with bipolar disorders. Child Abuse Negl..

[9] Montanari S., Terenzi B., Spera M.C., Donofrio G., Chieffo D.P.R., Monti L., Kotzalidis G.D., Sani G., Janiri D. (2025). Intergenerational transmission of childhood trauma in youths with mood disorders and their parents. J. Affect. Disord..

[10] Janiri D., Sani G., Rossi P., Piras F., Iorio M., Banaj N., Giuseppin G., Spinazzola E., Maggiora M., Ambrosi E. (2017). Amygdala and hippocampus volumes are differently affected by childhood trauma in patients with bipolar disorders and healthy controls. Bipolar Disord..

[11] Janiri D., Sani G., De Rossi P., Piras F., Banaj N., Ciullo V., Simonetti A., Arciniegas D.B., Spalletta G. (2019). Hippocampal subfield volumes and childhood trauma in bipolar disorders. J. Affect. Disord..

[12] Goldstein B.I. (2023). Comorbidity in pediatric bipolar disorder: An unmet challenge in need of treatment studies. Acta Psychiatr. Scand..

[13] Howland R.H. (2011). Update on newer antipsychotic drugs. J. Psychosoc. Nurs. Ment. Health Serv..

[14] Ishibashi T., Horisawa T., Tokuda K., Ishiyama T., Ogasa M., Tagashira R., Matsumoto K., Nishikawa H., Ueda Y., Toma S. (2010). Pharmacological profile of lurasidone, a novel antipsychotic agent with potent 5-hydroxytryptamine 7 (5-HT7) and 5-HT1A receptor activity. J. Pharmacol. Exp. Ther..

[15] Wróbel M.Z., Chodkowski A., Herold F., Marciniak M., Dawidowski M., Siwek A., Starowicz G., Stachowicz K., Szewczyk B., Nowak G. (2019). Synthesis and biological evaluation of new multi-target 3-(1H-indol-3-yl)pyrrolidine-2,5-dione derivatives with potential antidepressant effect. Eur. J. Med. Chem..

[16] Loebel A., Cucchiaro J., Silva R., Kroger H., Hsu J., Sarma K., Sachs G. (2014). Lurasidone monotherapy in the treatment of bipolar I depression: A randomized, double-blind, placebo-controlled study. Am. J. Psychiatry.

[17] Suppes T., Kroger H., Pikalov A., Loebel A. (2016). Lurasidone adjunctive with lithium or valproate for bipolar depression: A placebo-controlled trial utilizing prospective and retrospective enrolment cohorts. J. Psychiatr. Res..

[18] Ali Z., Tegin C., El-Mallakh R.S. (2020). Evaluating lurasidone as a treatment option for bipolar disorder. Expert Opin. Pharmacother..

[19] DelBello M.P., Goldman R., Phillips D., Deng L., Cucchiaro J., Loebel A. (2017). Efficacy and safety of lurasidone in children and adolescents with bipolar i depression: A double-blind, placebo-controlled study. J. Am. Acad. Child Adolesc. Psychiatry.

[20] Siafis S., Tzachanis D., Samara M., Papazisis G. (2018). Antipsychotic Drugs: From Receptor-binding Profiles to Metabolic Side Effects. Curr. Neuropharmacol..

[21] Findling R.L., Goldman R., Chiu Y.Y., Silva R., Jin F., Pikalov A., Loebel A. (2015). Pharmacokinetics and tolerability of lurasidone in children and adolescents with psychiatric disorders. Clin. Ther..

[22] Greenberg W.M., Citrome L. (2017). Pharmacokinetics and pharmacodynamics of lurasidone hydrochloride, a second-generation antipsychotic: A systematic review of the published literature. Clin. Pharmacokinet..

[23] Amerio A., Giacomini C., Fusar-Poli L., Aguglia A., Costanza A., Serafini G., Aguglia E., Amore M. (2021). Efficacy and safety of lurasidone in children and adolescents: Recommendations for clinical management and future research. Curr. Pharm. Des..

[24] Yatham L.N., Kennedy S.H., Parikh S.V., Schaffer A., Bond D.J., Frey B.N., Sharma V., Goldstein B.I., Rej S., Beaulieu S. (2018). Canadian Network for Mood and Anxiety Treatments (CANMAT) and International Society for Bipolar Disorders (ISBD) 2018 guidelines for the management of patients with bipolar disorder. Bipolar Disord..

[25] Singh M.K., Pikalov A., Siu C., Tocco M., Loebel A. (2020). Lurasidone in children and adolescents with bipolar depression presenting with mixed (subsyndromal hypomanic) features: *Post hoc* analysis of a randomized placebo-controlled trial. J. Child Adolesc. Psychopharmacol..

[26] DelBello M.P., Tocco M., Pikalov A., Deng L., Goldman R. (2021). Tolerability, safety, and effectiveness of two years of treatment with lurasidone in children and adolescents with bipolar depression. J. Child Adolesc. Psychopharmacol..

[27] Nair S.S., Chua C.J.M., Teo D.C.L. (2021). Lurasidone-induced manic switch in an adolescent with bipolar I disorder: A case report. East Asian Arch. Psychiatry.

[28] Page M.J., McKenzie J.E., Bossuyt P.M., Boutron I., Hoffmann T.C., Mulrow C.D., Shamseer L., Tetzlaff J.M., Akl E.A., Brennan S.E. (2021). The PRISMA 2020 statement: An updated guideline for reporting systematic reviews. BMJ.

[29] Moola S., Munn Z., Tufanaru C., Aromataris E., Sears K., Sfetcu R., Currie M., Qureshi R., Mattis P., Lisy K., Aromataris E., Munn Z. (2020). Chapter 7: Systematic reviews of etiology and risk. JBI Manual for Evidence Synthesis.

[30] Gagnier J.J., Kienle G., Altman D.G., Moher D., Sox H., Riley D., CARE Group (2013). The CARE guidelines: Consensus-based clinical case reporting guideline development. Headache.

[31] Sterne J.A.C., Savović J., Page M.J., Elbers R.G., Blencowe N.S., Boutron I., Cates C.J., Cheng H.-Y., Corbett M.S., Eldridge S.M. (2019). RoB 2: A revised tool for assessing risk of bias in randomised trials. BMJ.

[32] Channing J., Mitchell M., Cortese S. (2018). Lurasidone in children and adolescents: Systematic review and case report. J. Child Adolesc. Psychopharmacol..

[33] Prieto D.I., Zehgeer A.A., Connor D.F. (2019). Use of suvorexant for sleep regulation in an adolescent with early-onset bipolar disorder. J. Child Adolesc. Psychopharmacol..

[34] Mole T.B., Furlong Y., Clarke R.J., Rao P., Moore J.K., Pace G., Van Odyck H., Chen W. (2022). Lurasidone for adolescents with complex mental disorders: A case series. J. Pharm. Pract..

[35] Kadakia A., Dembek C., Liu Y., Dieyi C., Williams G.R. (2021). Hospitalization risk in pediatric patients with bipolar disorder treated with lurasidone vs. other oral atypical antipsychotics: A real-world retrospective claims database study. J. Med. Econ..

[36] Singh M.K., Siu C., Tocco M., Pikalov A., Loebel A. (2023). Sleep disturbance, irritability, and response to lurasidone treatment in children and adolescents with bipolar depression. Curr. Neuropharmacol..

[37] Diao X., Luo D., Wang D., Lai J., Li Q., Zhang P., Huang H., Wu L., Lu S., Hu S. (2022). Lurasidone versus Quetiapine for Cognitive Impairments in Young Patients with Bipolar Depression: A Randomized, Controlled Study. Pharmaceuticals.

[38] Janiri D., De Rossi P., Kotzalidis G.D., Girardi P., Koukopoulos A.E., Reginaldi D., Dotto F., Manfredi G., Jollant F., Gorwood P. (2018). Psychopathological characteristics and adverse childhood events are differentially associated with suicidal ideation and suicidal acts in mood disorders. Eur. Psychiatry.

[39] Janiri D., Di Luzio M., Montanari S., Hirsch D., Simonetti A., Moccia L., Conte E., Contaldo I., Veredice C., Mercuri E. (2024). Childhood trauma and self-harm in youths with bipolar disorders. Curr. Neuropharmacol..

[40] Corponi F., Fabbri C., Bitter I., Montgomery S., Vieta E., Kasper S., Pallanti S., Serretti A. (2019). Novel antipsychotics specificity profile: A clinically oriented review of lurasidone, brexpiprazole, cariprazine and lumateperone. Eur. Neuropsychopharmacol..

[41] Patel R.S., Veluri N., Patel J., Patel R., Machado T., Diler R. (2021). Second-generation antipsychotics in management of acute pediatric bipolar depression: A systematic review and meta-analysis. J. Child Adolesc. Psychopharmacol..

[42] DelBello M.P., Kadakia A., Heller V., Singh R., Hagi K., Nosaka T., Loebel A. (2022). Systematic review and network meta-analysis: Efficacy and safety of second-generation antipsychotics in youths with bipolar depression. J. Am. Acad. Child Adolesc. Psychiatry.

[43] Feng X.Z., Li Z., Li Z.Y., Wang K., Tan X., Zhao Y.Y., Mi W.F., Zhu W.L., Bao Y.P., Lu L. (2024). Effectiveness and safety of second-generation antipsychotics for psychiatric disorders apart from schizophrenia: A systematic review and meta-analysis. Psychiatry Res..

[44] Chakrabarti S. (2016). Treatment-adherence in bipolar disorder: A patient-centred approach. World J. Psychiatry.

[45] Pasternak B., Svanström H., Ranthe M.F., Melbye M., Hviid A. (2014). Atypical antipsychotics olanzapine, quetiapine, and risperidone and risk of acute major cardiovascular events in young and middle-aged adults: A nationwide register-based cohort study in Denmark. CNS Drugs.

[46] Rajagopalan K., Trueman D., Crowe L., Squirrell D., Loebel A. (2016). Cost-utility analysis of lurasidone versus aripiprazole in adults with schizophrenia. Pharmacoeconomics.

[47] Zyryanov S.K., Dyakov I.N., Juperin A.A., Egorova D.A., Mosolova E.S. (2020). Фармакoэкoнoмическая эффективнoсть применения препарата Луразидoн при лечении шизoфрении [The pharmacoeconomic efficacy of Lurasidone in the treatment of schizophrenia]. Zh. Nevrol. Psikhiatr. Im. S. S. Korsakova.

[48] Kato T., Ishigooka J., Miyajima M., Watabe K., Fujimori T., Masuda T., Higuchi T., Vieta E. (2020). Double-blind, placebo-controlled study of lurasidone monotherapy for the treatment of bipolar I depression. Psychiatry Clin. Neurosci..

[49] Reynolds G.P., Hill M.J., Kirk S.L. (2006). The 5-HT2C receptor and antipsychoticinduced weight gain–mechanisms and genetics. J. Psychopharmacol..

[50] Franklin R., Zorowitz S., Corse A.K., Widge A.S., Deckersbach T. (2015). Lurasidone for the treatment of bipolar depression: An evidence-based review. Neuropsychiatr. Dis. Treat..

[51] Halford J.C., Harrold J.A. (2012). 5-HT_2C_ receptor agonists and the control of appetite. Handb. Exp. Pharmacol..

[52] Caccia S., Pasina L., Nobili A. (2012). Critical appraisal of lurasidone in the management of schizophrenia. Neuropsychiatr. Dis. Treat..

[53] Kane J.M., Barnes T.R., Correll C.U., Sachs G., Buckley P., Eudicone J., McQuade R., Tran Q.V., Pikalov A., Assunção-Talbott S. (2010). Evaluation of akathisia in patients with schizophrenia, schizoaffective disorder, or bipolar I disorder: A post hoc analysis of pooled data from short- and long-term aripiprazole trials. J. Psychopharmacol..

[54] Salem H., Nagpal C., Pigott T., Teixeira A.L. (2017). Revisiting Antipsychotic-induced Akathisia: Current Issues and Prospective Challenges. Curr. Neuropharmacol..

[55] McIntyre R.S., Cucchiaro J., Pikalov A., Kroger H., Loebel A. (2015). Lurasidone in the treatment of bipolar depression with mixed (subsyndromal hypomanic) features: Post *hoc* analysis of a randomized placebo-controlled trial. J. Clin. Psychiatry.

[56] Danek P.J., Daniel W.A. (2022). The atypical antipsychotic lurasidone affects brain but not liver cytochrome P450 2D (CYP2D) activity. A comparison with other novel neuroleptics and significance for drug treatment of schizophrenia. Cells.

[57] Chen C.K., Wu L.S., Huang M.C., Kuo C.J., Cheng A.T. (2022). Antidepressant treatment and manic switch in bipolar I disorder: A clinical and molecular genetic study. J. Pers. Med..

[58] Vallabh A. (2024). Pharmacologic treatment of bipolar disorder and comorbid adult attention-deficit/hyperactivity disorder. Ment. Health Clin..

