# Supplementary material for: Lurasidone for Pediatric Bipolar Disorder: A Systematic Review
Source: Pharmaceuticals (Basel). 2025 Jun 30;18(7):979. doi: 10.3390/ph18070979 (PMC12298892; doi:10.3390/ph18070979)
Supplement: Supplementary file 1 [file pharmaceuticals-18-00979-s001.zip › pharmaceuticals-3667578-supplementary.pdf]

Supplementary Table S1. Records obtained in our PubMed, CINAHL, PsycINFO/PsycARTICLES, Scopus and ClinicalTrials.gov searches and decisions for eligibility or exclusion, with reasons for exclusion (3rd column).

PubMed lurasidone AND ("bipolar disorder" OR "bipolar depression" OR mani OR manic) filtered by age 0-18 years; species, humans, 11.6.2025 → 38 results		
1	Howland RH. Update on newer antipsychotic drugs. J Psychosoc Nurs Ment Health Serv. 2011;49(4):13-5. doi: 10.3928/02793695-20110311-99. Epub 2011 Mar 30.	Review
2	Loebel A, Cucchiaro J, Silva R, Kroger H, Hsu J, Sarma K, Sachs G. Lurasidone monotherapy in the treatment of bipolar I depression: a randomized, double- blind, placebo-controlled study. Am J Psychiatry. 2014;171(2):160-8. doi: 10.1176/appi.ajp.2013.13070984.	Adults
3	Loebel A, Cucchiaro J, Silva R, Kroger H, Sarma K, Xu J, Calabrese JR. Lurasidone as adjunctive therapy with lithium or valproate for the treatment of bipolar I depression: a randomized, double-blind, placebo-controlled study. Am J Psychiatry. 2014;171(2):169-77. doi: 10.1176/appi.ajp.2013.13070985.	Adults
4	Findling RL, Goldman R, Chiu YY, Silva R, Jin F, Pikalov A, Loebel A. Pharmacokinetics and Tolerability of Lurasidone in Children and Adolescents With Psychiatric Disorders. Clin Ther. 2015;37(12):2788-97. doi: 10.1016/j.clinthera.2015.11.001. Epub 2015 Nov 26.	Lumping
5	Suppes T, Kroger H, Pikalov A, Loebel A. Lurasidone adjunctive with lithium or valproate for bipolar depression: A placebo-controlled trial utilizing prospective and retrospective enrolment cohorts. J Psychiatr Res. 2016;78:86-93. doi: 10.1016/j.jpsychires.2016.03.012. Epub 2016 Mar 31.	Adult
6	McClellan J. Clinical Relevance Versus Statistical Significance. J Am Acad Child Adolesc Psychiatry. 2017;56(12):1008-1009. doi: 10.1016/j.jaac.2017.09.431.	Opinion
**7**	**DelBello MP, Goldman R, Phillips D, Deng L, Cucchiaro J, Loebel A. Efficacy and Safety of Lurasidone in Children and Adolescents With Bipolar I Depression: A Double-Blind, Placebo-Controlled Study. J Am Acad Child Adolesc Psychiatry. 2017;56(12):1015-1025. doi: 10.1016/j.jaac.2017.10.006. Epub 2017 Oct 13.**	**Included**
8	Yatham LN, Kennedy SH, Parikh SV, Schaffer A, Bond DJ, Frey BN, Sharma V, Goldstein BI, Rej S, Beaulieu S, Alda M, MacQueen G, Milev RV, Ravindran A, O'Donovan C, McIntosh D, Lam RW, Vazquez G, Kapczinski F, McIntyre RS, Kozicky J, Kanba S, Lafer B, Suppes T, Calabrese JR, Vieta E, Malhi G, Post RM, Berk M. Canadian Network for Mood and Anxiety Treatments (CANMAT) and International Society for Bipolar Disorders (ISBD) 2018 guidelines for the management of patients with bipolar disorder. Bipolar Disord. 2018;20(2):97-170. doi: 10.1111/bdi.12609. Epub 2018 Mar 14.	Review
9	Aman MG, Arnold LE, Barterian JA. Clinical Relevance Versus Statistical Significance: Aman and Colleagues Respond to Editorial. J Am Acad Child Adolesc Psychiatry. 2018;57(5):352-353. doi: 10.1016/j.jaac.2017.12.012.	Opinion
10	DelBello MP, Goldman R, Loebel A. Clinical Relevance Versus Statistical Significance: DelBello and Colleagues Respond to Editorial. J Am Acad Child Adolesc Psychiatry. 2018;57(5):353-354. doi: 10.1016/j.jaac.2018.01.024.	Opinion
**11**	**Channing J, Mitchell M, Cortese S. Lurasidone in Children and Adolescents: Systematic Review and Case Report. J Child Adolesc Psychopharmacol. 2018;28(7):428-436. doi: 10.1089/cap.2018.0046. Epub 2018 Jul 13.**	**Case**
12	Lee ES, Vidal C, Findling RL. A Focused Review on the Treatment of Pediatric Patients with Atypical Antipsychotics. J Child Adolesc Psychopharmacol. 2018;28(9):582-605. doi: 10.1089/cap.2018.0037. Epub 2018 Oct 12.	Review
**13**	**Prieto DI, Zehgeer AA, Connor DF. Use of Suvorexant for Sleep Regulation in an Adolescent with Early-Onset Bipolar Disorder. J Child Adolesc Psychopharmacol. 2019;29(5):395. doi: 10.1089/cap.2019.0029. Epub 2019 Apr 26.**	**Case**
14	Sun AY, Woods S, Findling RL, Stepanova E. Safety considerations in the psychopharmacology of pediatric bipolar disorder. Expert Opin Drug Saf. 2019;18(9):777-794. doi: 10.1080/14740338.2019.1637416. Epub 2019 Jul 11.	Review
15	Raison CL, Siu C, Pikalov A, Tocco M, Loebel A. C-reactive protein and response to lurasidone treatment in children and adolescents with bipolar I depression: Results from a placebo-controlled trial. Brain Behav Immun. 2020;84:269-274. doi: 10.1016/j.bbi.2019.12.010. Epub 2019 Dec 16.	Off-target
16	Weiss SJ, Cueto-Vilorio VA, Dharmaraj R, Barolia D, Nashat A, Walsh SJ, Simpson SE. Characterization of intentional lurasidone ingestions using the United States National Poison Data System. Clin Toxicol (Phila). 2020;58(12):1342-1346. doi: 10.1080/15563650.2020.1737102. Epub 2020 Mar 13.	Lumping
**17**	**Singh MK, Pikalov A, Siu C, Tocco M, Loebel A. Lurasidone in Children and Adolescents with Bipolar Depression Presenting with Mixed (Subsyndromal Hypomanic) Features: *Post Hoc* Analysis of a Randomized Placebo-Controlled Trial. J Child Adolesc Psychopharmacol. 2020;30(10):590-598. doi: 10.1089/cap.2020.0018. Epub 2020 May 8.**	**Included**
18	Kato T, Ishigooka J, Miyajima M, Watabe K, Fujimori T, Masuda T, Higuchi T, Vieta E. Double-blind, placebo-controlled study of lurasidone monotherapy for the treatment of bipolar I depression. Psychiatry Clin Neurosci. 2020;74(12):635-644. doi: 10.1111/pcn.13137. Epub 2020 Sep 24.	Adults
19	Post RM, Rowe M, Findling R. Little Agreement on Treating Residual Bipolar Disorder Symptoms in a Child. Prim Care Companion CNS Disord. 2020;22(5):19m02574. doi: 10.4088/PCC.19m02574.	Opinion
**20**	**Mole TB, Furlong Y, Clarke RJ, Rao P, Moore JK, Pace G, Van Odyck H, Chen W. Lurasidone for Adolescents With Complex Mental Disorders: A Case Series. J Pharm Pract. 2022;35(5):800-804. doi: 10.1177/0897190021997011. Epub 2021 Mar 24.**	**Case**
21	Keramatian K, Chakrabarty T, Saraf G, Yatham LN. New Developments in the Use of Atypical Antipsychotics in the Treatment of Bipolar Disorder: a Systematic Review of Recent Randomized Controlled Trials. Curr Psychiatry Rep. 2021;23(7):39. doi: 10.1007/s11920-021-01252-w.	Review
22	Post RM, Grunze H. The Challenges of Children with Bipolar Disorder. Medicina (Kaunas). 2021;57(6):601. doi: 10.3390/medicina57060601.	Opinion
**23**	**DelBello MP, Tocco M, Pikalov A, Deng L, Goldman R. Tolerability, Safety, and Effectiveness of Two Years of Treatment with Lurasidone in Children and Adolescents with Bipolar Depression. J Child Adolesc Psychopharmacol. 2021;31(7):494-503. doi: 10.1089/cap.2021.0040. Epub 2021 Jul 29.**	**Included**
24	Amerio A, Giacomini C, Fusar-Poli L, Aguglia A, Costanza A, Serafini G, Aguglia E, Amore M. Efficacy and Safety of Lurasidone in Children and Adolescents: Recommendations for Clinical Management and Future Research. Curr Pharm Des. 2021;27(39):4062-4069. doi: 10.2174/1381612827666210804110853.	Review
25	DelBello MP, Kadakia A, Heller V, Singh R, Hagi K, Nosaka T, Loebel A. Systematic Review and Network Meta-analysis: Efficacy and Safety of Second- Generation Antipsychotics in Youths With Bipolar Depression. J Am Acad Child Adolesc Psychiatry. 2022;61(2):243-254. doi: 10.1016/j.jaac.2021.03.021. Epub 2021 May 4.	Review
**26**	**Nair SS, Chua CJM, Teo DCL. Lurasidone-Induced Manic Switch in an Adolescent with Bipolar I Disorder: a Case Report. East Asian Arch Psychiatry. 2021;31(3):81-83. doi: 10.12809/eaap2040.**	**Case**
**27**	**Kadakia A, Dembek C, Liu Y, Dieyi C, Williams GR. Hospitalization risk in pediatric patients with bipolar disorder treated with lurasidone vs. other oral atypical antipsychotics: a real-world retrospective claims database study. J Med Econ. 2021;24(1):1212-1220. doi: 10.1080/13696998.2021.1993862.**	**Included**
28	Patel RS, Veluri N, Patel J, Patel R, Machado T, Diler R. Second-Generation Antipsychotics in Management of Acute Pediatric Bipolar Depression: A Systematic Review and Meta-analysis. J Child Adolesc Psychopharmacol. 2021;31(8):521-530. doi: 10.1089/cap.2021.0031.	Review
29	Goryunov AV. Горюнов А.В. Применение Луразидона при лечении психических заболеваний в детском возрасте [Using Lurasidone in the treatment of mental illness in childhood]. Zh Nevrol Psikhiatr Im S S Korsakova- Журнал неврологии и психиатрии им. С.С. Корсакова. 2021; т. 121, №11, вып. 2, с. 77–85. Russian. doi: 10.17116/jnevro202112111277.	Review

30	Anmella G, Pacchiarotti I, Hidalgo-Mazzei D, Fico G, Murru A, Sagué- Vilavella M, Amoretti S, Verdolini N, Radua J, Vieta E. Lamotrigine-induced mania: warning report for the identification of vulnerable populations and expert clinical recommendations for prescription. Int Clin Psychopharmacol. 2022;37(6):276-278. doi: 10.1097/YIC.0000000000000390. Epub 2022 Jan 31.	Off-target
31	Croatto G, Vancampfort D, Miola A, Olivola M, Fiedorowicz JG, Firth J, Alexinschi O, Gaina MA, Makkai V, Soares FC, Cavaliere L, Vianello G, Stubbs B, Fusar-Poli P, Carvalho AF, Vieta E, Cortese S, Shin JI, Correll CU, Solmi M. The impact of pharmacological and non-pharmacological interventions on physical health outcomes in people with mood disorders across the lifespan: An umbrella review of the evidence from randomised controlled trials. Mol Psychiatry. 2023;28(1):369-390. doi: 10.1038/s41380-022-01770-w. Epub 2022 Sep 22.	Off-target
**32**	**Singh MK, Siu C, Tocco M, Pikalov A, Loebel A. Sleep Disturbance, Irritability, and Response to Lurasidone Treatment in Children and Adolescents with Bipolar Depression. Curr Neuropharmacol. 2023;21(6):1393-1404. doi: 10.2174/1570159X20666220927112625.**	**Included**
33	Hobbs E, Reed R, Lorberg B, Robb AS, Dorfman J. Psychopharmacological Treatment Algorithms of Manic/Mixed and Depressed Episodes in Pediatric Bipolar Disorder. J Child Adolesc Psychopharmacol. 2022;32(10):507-521. doi: 10.1089/cap.2022.0035. Epub 2022 Dec 2.	Review
34	Ricci V, Martinotti G, De Berardis D, Maina G. Lurasidone use in Cannabis-Induced Psychosis: A Novel Therapeutic Strategy and Clinical Considerations in Four Cases Report. Int J Environ Res Public Health. 2022;19(23):16057. doi: 10.3390/ijerph192316057.	Adult
35	Garcia-Rodriguez L, Burton DJ, Leonards CA, Davey CG. Effectiveness of atypical antipsychotics for unipolar and bipolar depression in adolescents and young adults: A systematic review and meta-analysis. J Affect Disord. 2023;339:633-639. doi: 10.1016/j.jad.2023.07.082. Epub 2023 Jul 17.	Review
36	Solmi M, De Toffol M, Kim JY, Choi MJ, Stubbs B, Thompson T, Firth J, Miola A, Croatto G, Baggio F, Michelon S, Ballan L, Gerdle B, Monaco F, Simonato P, Scocco P, Ricca V, Castellini G, Fornaro M, Murru A, Vieta E, Fusar-Poli P, Barbui C, Ioannidis JPA, Carvalho AF, Radua J, Correll CU, Cortese S, Murray RM, Castle D, Shin JI, Dragioti E. Balancing risks and benefits of cannabis use: umbrella review of meta-analyses of randomised controlled trials and observational studies. BMJ. 2023;382:e072348. doi: 10.1136/bmj-2022-072348.	Off-target
37	Amerio A, Arduino G, Fesce F, Costanza A, Aguglia A, Amore M, Serafini G. Advances in the management of bipolar disorder in children and adolescents: an update on the literature. Expert Rev Neurother. 2024;24(10):1011-1024. doi: 10.1080/14737175.2024.2386429. Epub 2024 Aug 5.	Review
38	Kernizan N, Forinash A, Yancey A, Kruger S, Chavan NR, Mathews K. Mood stabilizers for treatment of bipolar disorder in pregnancy and impact on neonatal outcomes. Bipolar Disord. 2024;26(8):779-784. doi: 10.1111/bdi.13481. Epub 2024 Aug 22.	Off-target
CINAHL 11.6.2025	lurasidone AND bipolar disorder AND (pediatric or child or children or infant or adolescent) → 22 results	
39	Howland RH. Update on newer antipsychotic drugs. J Psychosoc Nurs Ment Health Serv. 2011;49(4):13-5. doi: 10.3928/02793695-20110311-99. Epub 2011 Mar 30.	Dupl. 1PM
40	De Hert M, Yu W, Detraux J, Sweers K, van Winkel R, Correll CU. Body weight and metabolic adverse effects of asenapine, iloperidone, lurasidone and paliperidone in the treatment of schizophrenia and bipolar disorder: a systematic review and exploratory meta-analysis. CNS Drugs. 2012;26(9):733-59. doi: 10.2165/11634500-000000000-00000.	Review
41	Olanzapine-fluoxetine combination has some benefit, but placebo effect and adverse events high. The Brown University Child & Adolescent Psychopharmacology Update: Volume 17, Issue 4 pp. 1,7. First published: 29 March 2015 https://doi.org/10.1002/cpu.30031 Refers to Detke HC, DelBello MP, Landry J, Usher RW.Olanzapine/fluoxetine combination in childrenand adolescents with bipolar I depression: A randomized, double-blind, placebo-controlled trial.J Am Acad Child Adolesc Psychiatry 2015 Mar;54(3):217–224. doi: 10.1016/j.jaac.2014.12.012.	No lurasidone
42	DelBello MP, Goldman R, Phillips D, Deng L, Cucchiaro J, Loebel A. Efficacy and Safety of Lurasidone in Children and Adolescents With Bipolar I Depression: A Double-Blind, Placebo-Controlled Study. J Am Acad Child Adolesc Psychiatry. 2017;56(12):1015-1025. doi: 10.1016/j.jaac.2017.10.006. Epub 2017 Oct 13.	Dupl. 7PM
43	RCT finds lurasidone improves bipolar I depression in children and adolescents. The Brown University Child & Adolescent Psychopharmacology Update: Volume 20, Issue 2, pp. 1-3. First published: 30 January 2018 https://doi.org/10.1002/cpu.30272 Refers to DelBello MP, Goldman R, Phillips D, et al. Efficacy and safety of lurasidone in children and adolescents with bipolar I depression: A double-blind, placebo-controlled study. J Am Acad Child Adolesc Psychia-try 2017; 56(12):1015-25. doi: 10.1016/j.jaac.2017.10.006. Epub 2017 Oct 13.	Dupl. 7PM
44	DelBello MP, Goldman R, Loebel A. Clinical Relevance Versus Statistical Significance: DelBello and Colleagues Respond to Editorial. J Am Acad Child Adolesc Psychiatry. 2018;57(5):353-354. doi: 10.1016/j.jaac.2018.01.024.	Dupl. 10PM
45	Channing J, Mitchell M, Cortese S. Lurasidone in Children and Adolescents: Systematic Review and Case Report. J Child Adolesc Psychopharmacol. 2018;28(7):428-436. doi: 10.1089/cap.2018.0046. Epub 2018 Jul 13.	Dupl. 11PM
46	Lee ES, Vidal C, Findling RL. A Focused Review on the Treatment of Pediatric Patients with Atypical Antipsychotics. J Child Adolesc Psychopharmacol. 2018;28(9):582-605. doi: 10.1089/cap.2018.0037. Epub 2018 Oct 12.	Dupl. 12PM
47	DelBello MP, Goldman R, Tocco M, Deng L, Pikalov A. 159 Safety and Efficacy of Lurasidone in Children and Adolescents with Bipolar Depression: Results from a 2-Year Open-label Extension Study. CNS Spectr. 2020;25(2):301-2. DOI: https://doi.org/10.1017/S1092852920000759. Later published as DelBello MP, Tocco M, Pikalov A, Deng L, Goldman R. Tolerability, Safety, and Effectiveness of Two Years of Treatment with Lurasidone in Children and Adolescents with Bipolar Depression. J Child Adolesc Psychopharmacol. 2021;31(7):494-503. doi: 10.1089/cap.2021.0040. Epub 2021 Jul 29. #23PM	Congr. Abstr.
48	Singh MK, Pikalov A, Siu C, Tocco M, Loebel A. Lurasidone in Children and Adolescents with Bipolar Depression Presenting with Mixed (Subsyndromal Hypomanic) Features: *Post Hoc* Analysis of a Randomized Placebo-Controlled Trial. J Child Adolesc Psychopharmacol. 2020;30(10):590-598. doi: 10.1089/cap.2020.0018. Epub 2020 May 8.	Dupl. 17PM
49	Kadakia A, Dembek C, Liu Y, Dieyi C, Williams GR. Hospitalization risk in pediatric patients with bipolar disorder treated with lurasidone vs. other oral atypical antipsychotics: a real-world retrospective claims database study. J Med Econ. 2021;24(1):1212-1220. doi: 10.1080/13696998.2021.1993862.	Dupl. 27PM
50	Singh M, Tocco M, Schweizer E, Pikalov A. Long-Term Effectiveness of Lurasidone in Pediatric Bipolar Depression: Response, Remission and Recovery. CNS Spectr. 2021;26(2):148. DOI: https://doi.org/10.1017/S1092852920002345.	Congr. Abstr.
51	Keramatian K, Chakrabarty T, Saraf G, Yatham LN. New Developments in the Use of Atypical Antipsychotics in the Treatment of Bipolar Disorder: a Systematic Review of Recent Randomized Controlled Trials. Curr Psychiatry Rep. 2021;23(7):39. doi: 10.1007/s11920-021-01252-w.	Dupl. 21PM
52	Nair SS, Chua CJM, Teo DCL. Lurasidone-Induced Manic Switch in an Adolescent with Bipolar I Disorder: a Case Report. East Asian Arch Psychiatry. 2021;31(3):81-83. doi: 10.12809/eaap2040.	Dupl. 26PM
53	DelBello MP, Tocco M, Pikalov A, Deng L, Goldman R. Tolerability, Safety, and Effectiveness of Two Years of Treatment with Lurasidone in Children and Adolescents with Bipolar Depression. J Child Adolesc Psychopharmacol. 2021;31(7):494-503. doi: 10.1089/cap.2021.0040. Epub 2021 Jul 29.	Dupl. 23PM
54	Knopf A. Lurasidone found effective and safe for children with bipolar depression. Brown University Child & Adolescent Psychopharmacology Update. 2021;23(10):1-3. First published 13 September 2021 https://doi.org/10.1002/cpu.30612. Refers to DelBello MP, Tocco M, Pikalov A, et al. Tolerability, safety, and effectiveness of two years of treatment with lurasidone in children and adolescents with bipolar depression. J Child Adolesc Psychopharmcol 2021 Jul 29; doi: 10.1089/cap.2021.0040. #23PM	Dupl. 23PM
55	Patel RS, Veluri N, Patel J, Patel R, Machado T, Diler R. Second-Generation Antipsychotics in Management of Acute Pediatric Bipolar Depression: A Systematic Review and Meta-analysis. J Child Adolesc Psychopharmacol. 2021;31(8):521-530. doi: 10.1089/cap.2021.0031.	Dupl. 28PM

56	Knopf A. Lurasidone found effective and safe for children with bipolar depression. The Brown University Child and Adolescent Behavior Letter. 2021;37(11):6-7. Refers to DelBello MP, Tocco M, Pikalov A, et al. Tolerability, safety, and effectiveness of two years of treatment with lurasidone in children and adolescents with bipolar depression. J Child Adolesc Psychopharmcol 2021 Jul 29; doi: 10.1089/cap.2021.0040. #23PM	Dupl. 23PM
57	Kadakia A, Dembek C, Liu Y, Dieyi C, Williams GR. Hospitalization risk in pediatric patients with bipolar disorder treated with lurasidone vs. other oral atypical antipsychotics: a real-world retrospective claims database study. J Med Econ. 2021;24(1):1212-1220. doi: 10.1080/13696998.2021.1993862.	Dupl. 27PM 11C
58	DelBello MP, Kadakia A, Heller V, Singh R, Hagi K, Nosaka T, Loebel A. Systematic Review and Network Meta-analysis: Efficacy and Safety of Second-Generation Antipsychotics in Youths With Bipolar Depression. J Am Acad Child Adolesc Psychiatry. 2022;61(2):243-254. doi: 10.1016/j.jaac.2021.03.021. Epub 2021 May 4.	Review
59	Mole TB, Furlong Y, Clarke RJ, Rao P, Moore JK, Pace G, Van Odyck H, Chen W. Lurasidone for Adolescents With Complex Mental Disorders: A Case Series. J Pharm Pract. 2022;35(5):800-804. doi: 10.1177/0897190021997011. Epub 2021 Mar 24.	Dupl. 20PM
60	Hobbs E, Reed R, Lorberg B, Robb AS, Dorfman J. Psychopharmacological Treatment Algorithms of Manic/Mixed and Depressed Episodes in Pediatric Bipolar Disorder. J Child Adolesc Psychopharmacol. 2022;32(10):507-521. doi: 10.1089/cap.2022.0035. Epub 2022 Dec 2.	Dupl. 33PM
PsycINFO/PsycARTICLES 11.6.2025	lurasidone AND bipolar disorder AND (pediatric or child or children or infant or adolescent) → 23 results	
61	Howland RH. Update on newer antipsychotic drugs. J Psychosoc Nurs Ment Health Serv. 2011;49(4):13-5. doi: 10.3928/02793695-20110311-99. Epub 2011 Mar 30.	Dupl. 1PM 1C
62	Bipolar Disord. 2013;15(s1):1-163. Special Issue:Abstracts of the Tenth International Conference on Bipolar Disorder, 13–16 June, 2013, Miami Beach, Florida, USA.	Congr. Abstr.
63	Loebel A, Cucchiaro J, Silva R, Kroger H, Hsu J, Sarma K, Sachs G. Lurasidone monotherapy in the treatment of bipolar I depression: a randomized, double- blind, placebo-controlled study. Am J Psychiatry. 2014;171(2):160-8. doi: 10.1176/appi.ajp.2013.13070984.	Dupl. 2PM
64	Loebel A, Cucchiaro J, Silva R, Kroger H, Sarma K, Xu J, Calabrese JR. Lurasidone as adjunctive therapy with lithium or valproate for the treatment of bipolar I depression: a randomized, double-blind, placebo-controlled study. Am J Psychiatry. 2014;171(2):169-77. doi: 10.1176/appi.ajp.2013.13070985.	Dupl. 3PM
65	DelBello MP, Goldman R, Phillips D, Deng L, Cucchiaro J, Loebel A. Efficacy and Safety of Lurasidone in Children and Adolescents With Bipolar I Depression: A Double-Blind, Placebo-Controlled Study. J Am Acad Child Adolesc Psychiatry. 2017;56(12):1015-1025. doi: 10.1016/j.jaac.2017.10.006. Epub 2017 Oct 13.	Dupl. 7PM 4C 5C
66	Yatham LN, Kennedy SH, Parikh SV, Schaffer A, Bond DJ, Frey BN, Sharma V, Goldstein BI, Rej S, Beaulieu S, Alda M, MacQueen G, Milev RV, Ravindran A, O'Donovan C, McIntosh D, Lam RW, Vazquez G, Kapczinski F, McIntyre RS, Kozicky J, Kanba S, Lafer B, Suppes T, Calabrese JR, Vieta E, Malhi G, Post RM, Berk M. Canadian Network for Mood and Anxiety Treatments (CANMAT) and International Society for Bipolar Disorders (ISBD) 2018 guidelines for the management of patients with bipolar disorder. Bipolar Disord. 2018;20(2):97-170. doi: 10.1111/bdi.12609. Epub 2018 Mar 14.	Dupl. 8PM
67	DelBello MP, Goldman R, Loebel A. Clinical Relevance Versus Statistical Significance: DelBello and Colleagues Respond to Editorial. J Am Acad Child Adolesc Psychiatry. 2018;57(5):353-354. doi: 10.1016/j.jaac.2018.01.024.	Dupl. 10PM 6C
68	Channing J, Mitchell M, Cortese S. Lurasidone in Children and Adolescents: Systematic Review and Case Report. J Child Adolesc Psychopharmacol. 2018;28(7):428-436. doi: 10.1089/cap.2018.0046. Epub 2018 Jul 13.	Dupl. 11PM 7C
69	Lee ES, Vidal C, Findling RL. A Focused Review on the Treatment of Pediatric Patients with Atypical Antipsychotics. J Child Adolesc Psychopharmacol. 2018;28(9):582-605. doi: 10.1089/cap.2018.0037. Epub 2018 Oct 12.	Dupl. 12PM 8C
70	Prieto DI, Zehgeer AA, Connor DF. Use of Suvorexant for Sleep Regulation in an Adolescent with Early-Onset Bipolar Disorder. J Child Adolesc Psychopharmacol. 2019;29(5):395. doi: 10.1089/cap.2019.0029. Epub 2019 Apr 26.	Dupl. 13PM
71	Raison CL, Siu C, Pikalov A, Tocco M, Loebel A. C-reactive protein and response to lurasidone treatment in children and adolescents with bipolar I depression: Results from a placebo-controlled trial. Brain Behav Immun. 2020;84:269-274. doi: 10.1016/j.bbi.2019.12.010. Epub 2019 Dec 16.	Dupl. 15PM
72	Singh MK, Pikalov A, Siu C, Tocco M, Loebel A. Lurasidone in Children and Adolescents with Bipolar Depression Presenting with Mixed (Subsyndromal Hypomanic) Features: *Post Hoc* Analysis of a Randomized Placebo-Controlled Trial. J Child Adolesc Psychopharmacol. 2020;30(10):590-598. doi: 10.1089/cap.2020.0018. Epub 2020 May 8.	Dupl. 17PM 10C
73	Nair SS, Chua CJM, Teo DCL. Lurasidone-Induced Manic Switch in an Adolescent with Bipolar I Disorder: a Case Report. East Asian Arch Psychiatry. 2021;31(3):81-83. doi: 10.12809/eaap2040.	Dupl. 26PM 15C
74	DelBello MP, Tocco M, Pikalov A, Deng L, Goldman R. Tolerability, Safety, and Effectiveness of Two Years of Treatment with Lurasidone in Children and Adolescents with Bipolar Depression. J Child Adolesc Psychopharmacol. 2021;31(7):494-503. doi: 10.1089/cap.2021.0040. Epub 2021 Jul 29.	Dupl. 23PM 15C
75	Patel RS, Veluri N, Patel J, Patel R, Machado T, Diler R. Second-Generation Antipsychotics in Management of Acute Pediatric Bipolar Depression: A Systematic Review and Meta-analysis. J Child Adolesc Psychopharmacol. 2021;31(8):521-530. doi: 10.1089/cap.2021.0031.	Dupl. 28PM 17C
76	Janas-Kozik M, Dudek D, Heitzman J, Remberk B, Samochowiec J, Słopień A, Wichniak A. Polish Psychiatric Association diagnostic and therapeutic management guidelines for patients with early-onset schizophrenia. Psychiatr Pol. 2022 Aug 31;56(4):675-695. English, Polish. doi: 10.12740/PP/OnlineFirst/149707. Epub 2022 Aug 31.	Review
77	DelBello MP, Kadakia A, Heller V, Singh R, Hagi K, Nosaka T, Loebel A. Systematic Review and Network Meta-analysis: Efficacy and Safety of Second-Generation Antipsychotics in Youths With Bipolar Depression. J Am Acad Child Adolesc Psychiatry. 2022;61(2):243-254. doi: 10.1016/j.jaac.2021.03.021. Epub 2021 May 4.	Dupl. 20C
78	Hobbs E, Reed R, Lorberg B, Robb AS, Dorfman J. Psychopharmacological Treatment Algorithms of Manic/Mixed and Depressed Episodes in Pediatric Bipolar Disorder. J Child Adolesc Psychopharmacol. 2022;32(10):507-521. doi: 10.1089/cap.2022.0035. Epub 2022 Dec 2.	Dupl. 33PM 22C
79	Croatto G, Vancampfort D, Miola A, Olivola M, Fiedorowicz JG, Firth J, Alexinschi O, Gaina MA, Makkai V, Soares FC, Cavaliere L, Vianello G, Stubbs B, Fusar-Poli P, Carvalho AF, Vieta E, Cortese S, Shin JI, Correll CU, Solmi M. The impact of pharmacological and non-pharmacological interventions on physical health outcomes in people with mood disorders across the lifespan: An umbrella review of the evidence from randomised controlled trials. Mol Psychiatry. 2023;28(1):369-390. doi: 10.1038/s41380-022-01770-w. Epub 2022 Sep 22.	Dupl. 31PM
80	Singh MK, Siu C, Tocco M, Pikalov A, Loebel A. Sleep Disturbance, Irritability, and Response to Lurasidone Treatment in Children and Adolescents with Bipolar Depression. Curr Neuropharmacol. 2023;21(6):1393-1404. doi: 10.2174/1570159X20666220927112625.	Dupl. 32PM
81	Cohen LS, Church TR, Freeman MP, Gaccione P, Caplin PS, Kobylski LA, Arakelian M, Rossa ET, Chitayat D, Hernández-Díaz S, Viguera AC. Reproductive Safety of Lurasidone and Quetiapine: Update from the National Pregnancy Registry for Psychiatric Medications. J Womens Health (Larchmt). 2023;32(4):452-462. doi: 10.1089/jwh.2022.0310. Epub 2023 Jan 30.	Off-target
82	Garcia-Rodriguez L, Burton DJ, Leonards CA, Davey CG. Effectiveness of atypical antipsychotics for unipolar and bipolar depression in adolescents and young adults: A systematic review and meta-analysis. J Affect Disord. 2023;339:633-639. doi: 10.1016/j.jad.2023.07.082. Epub 2023 Jul 17.	Dupl. 35PM
83	Amerio A, Arduino G, Fesce F, Costanza A, Aguglia A, Amore M, Serafini G. Advances in the management of bipolar disorder in children and adolescents: an update on the literature. Expert Rev Neurother. 2024;24(10):1011-1024. doi: 10.1080/14737175.2024.2386429. Epub 2024 Aug 5.	Dupl. 37PM
Scopus 11.6.2025	lurasidone[title] AND bipolar disorder[title, abstract, keywords] AND (children OR adolescents) [title, abstract, keywords] → 24 results	

84	De Hert M, Yu W, Detraux J, Sweers K, van Winkel R, Correll CU. Body weight and metabolic adverse effects of asenapine, iloperidone, lurasidone and paliperidone in the treatment of schizophrenia and bipolar disorder: a systematic review and exploratory meta-analysis. CNS Drugs. 2012;26(9):733-59. doi: 10.2165/11634500-000000000-00000.	Dupl. C2
85	Loebel A, Cucchiaro J, Silva R, Kroger H, Sarma K, Xu J, Calabrese JR. Lurasidone as adjunctive therapy with lithium or valproate for the treatment of bipolar I depression: a randomized, double-blind, placebo-controlled study. Am J Psychiatry. 2014;171(2):169-77. doi: 10.1176/appi.ajp.2013.13070985.	Dupl. 3PM 4Ps
86	Loebel A, Cucchiaro J, Silva R, Kroger H, Hsu J, Sarma K, Sachs G. Lurasidone monotherapy in the treatment of bipolar I depression: a randomized, double- blind, placebo-controlled study. Am J Psychiatry. 2014;171(2):160-8. doi: 10.1176/appi.ajp.2013.13070984.	Dupl. 2PM 3Ps
87	Nasrallah HA, Cucchiaro JB, Mao Y, Pikalov AA, Loebel AD. Lurasidone for the treatment of depressive symptoms in schizophrenia: analysis of 4 pooled, 6-week, placebo-controlled studies. CNS Spectr. 2015;20(2):140-7. doi: 10.1017/S1092852914000285. Epub 2014 Jun 23.	No BD
88	Findling RL, Goldman R, Chiu YY, Silva R, Jin F, Pikalov A, Loebel A. Pharmacokinetics and Tolerability of Lurasidone in Children and Adolescents With Psychiatric Disorders. Clin Ther. 2015;37(12):2788-97. doi: 10.1016/j.clinthera.2015.11.001. Epub 2015 Nov 26.	Dupl. 4PM
89	Suppes T, Kroger H, Pikalov A, Loebel A. Lurasidone adjunctive with lithium or valproate for bipolar depression: A placebo-controlled trial utilizing prospective and retrospective enrolment cohorts. J Psychiatr Res. 2016;78:86-93. doi: 10.1016/j.jpsychires.2016.03.012. Epub 2016 Mar 31.	Dupl. 5PM
90	DelBello MP, Goldman R, Phillips D, Deng L, Cucchiaro J, Loebel A. Efficacy and Safety of Lurasidone in Children and Adolescents With Bipolar I Depression: A Double-Blind, Placebo-Controlled Study. J Am Acad Child Adolesc Psychiatry. 2017;56(12):1015-1025. doi: 10.1016/j.jaac.2017.10.006. Epub 2017 Oct 13.	Dupl. 7PM 4C 5C 5Ps
91	Rafi M, Goyal C, Reddy P, Reddy S. Lurasidone Induced Thrombocytopenia: Is it a Signal of Drug Induced Myelosuppression? Indian J Psychol Med. 2018;40(2):191-192. doi: 10.4103/IJPSYM.IJPSYM_374_17.	Adult
92	Osborne IJ, Mace S, Taylor D. A prospective year-long follow-up of lurasidone use in clinical practice: factors predicting treatment persistence. Ther Adv Psychopharmacol. 2018 Apr;8(4):117-125. doi: 10.1177/2045125317749740. Epub 2018 Jan 7.	Adult
93	Channing J, Mitchell M, Cortese S. Lurasidone in Children and Adolescents: Systematic Review and Case Report. J Child Adolesc Psychopharmacol. 2018;28(7):428-436. doi: 10.1089/cap.2018.0046. Epub 2018 Jul 13.	Dupl. 11PM 7C 8Ps
94	Weiss SJ, Cueto-Vilorio VA, Dharmaraj R, Barolia D, Nashat A, Walsh SJ, Simpson SE. Characterization of intentional lurasidone ingestions using the United States National Poison Data System. Clin Toxicol (Phila). 2020;58(12):1342-1346. doi: 10.1080/15563650.2020.1737102. Epub 2020 Mar 13.	Dupl. 16PM
95	Raison CL, Siu C, Pikalov A, Tocco M, Loebel A. C-reactive protein and response to lurasidone treatment in children and adolescents with bipolar I depression: Results from a placebo-controlled trial. Brain Behav Immun. 2020;84:269-274. doi: 10.1016/j.bbi.2019.12.010. Epub 2019 Dec 16.	Dupl. 15PM 11Ps
96	Kato T, Ishigooka J, Miyajima M, Watabe K, Fujimori T, Masuda T, Higuchi T, Vieta E. Double-blind, placebo-controlled study of lurasidone monotherapy for the treatment of bipolar I depression. Psychiatry Clin Neurosci. 2020;74(12):635-644. doi: 10.1111/pcn.13137. Epub 2020 Sep 24.	Dupl. 18PM
97	Singh MK, Pikalov A, Siu C, Tocco M, Loebel A. Lurasidone in Children and Adolescents with Bipolar Depression Presenting with Mixed (Subsyndromal Hypomanic) Features: Post Hoc Analysis of a Randomized Placebo-Controlled Trial. J Child Adolesc Psychopharmacol. 2020;30(10):590-598. doi: 10.1089/cap.2020.0018. Epub 2020 May 8.	Dupl. 17PM 10C 12Ps
98	Goryunov AV. Горюнов А.В. Применение Луразидона при лечении психических заболеваний в детском возрасте [Using Lurasidone in the treatment of mental illness in childhood]. Zh Nevrol Psikhiatr Im S S Korsakova- Журнал неврологии и психиатрии им. С.С. Корсакова. 2021; т. 121, №11, вып. 2, с. 77–85. Russian. doi: 10.17116/jnevro202112111277.	Dupl. 29PM
99	Amerio A, Giacomini C, Fusar-Poli L, Aguglia A, Costanza A, Serafini G, Aguglia E, Amore M. Efficacy and Safety of Lurasidone in Children and Adolescents: Recommendations for Clinical Management and Future Research. Curr Pharm Des. 2021;27(39):4062-4069. doi: 10.2174/1381612827666210804110853.	Dupl. 24PM
100	Kadakia A, Dembek C, Liu Y, Dieyi C, Williams GR. Hospitalization risk in pediatric patients with bipolar disorder treated with lurasidone vs. other oral atypical antipsychotics: a real-world retrospective claims database study. J Med Econ. 2021;24(1):1212-1220. doi: 10.1080/13696998.2021.1993862.	Dupl. 27PM 11C 19C
101	Nair SS, Chua CJM, Teo DCL. Lurasidone-Induced Manic Switch in an Adolescent with Bipolar I Disorder: a Case Report. East Asian Arch Psychiatry. 2021;31(3):81-83. doi: 10.12809/eaap2040.	Dupl. 26PM 15C 13Ps
102	DelBello MP, Tocco M, Pikalov A, Deng L, Goldman R. Tolerability, Safety, and Effectiveness of Two Years of Treatment with Lurasidone in Children and Adolescents with Bipolar Depression. J Child Adolesc Psychopharmacol. 2021;31(7):494-503. doi: 10.1089/cap.2021.0040. Epub 2021 Jul 29.	Dupl. 23PM 15C 14Ps
103	Naguy A, Al-Khadhari S, Pridmore S. Possible Lurasidone-Associated Dose-Dependent QTc Prolongation in First-Episode Psychosis. Psychopharmacol Bull. 2022;52(3):68-71.	No BD
104	Mole TB, Furlong Y, Clarke RJ, Rao P, Moore JK, Pace G, Van Odyck H, Chen W. Lurasidone for Adolescents With Complex Mental Disorders: A Case Series. J Pharm Pract. 2022;35(5):800-804. doi: 10.1177/0897190021997011. Epub 2021 Mar 24.	Dupl. 20PM 22C
**105**	**Diao X, Luo D, Wang D, Lai J, Li Q, Zhang P, Huang H, Wu L, Lu S, Hu S. Lurasidone versus Quetiapine for Cognitive Impairments in Young Patients with Bipolar Depression: A Randomized, Controlled Study. Pharmaceuticals (Basel). 2022;15(11):1403. doi: 10.3390/ph15111403.**	**Included**
106	Ricci V, Martinotti G, De Berardis D, Maina G. Lurasidone use in Cannabis-Induced Psychosis: A Novel Therapeutic Strategy and Clinical Considerations in Four Cases Report. Int J Environ Res Public Health. 2022;19(23):16057. doi: 10.3390/ijerph192316057.	Dupl. 34PM
107	Singh MK, Siu C, Tocco M, Pikalov A, Loebel A. Sleep Disturbance, Irritability, and Response to Lurasidone Treatment in Children and Adolescents with Bipolar Depression. Curr Neuropharmacol. 2023;21(6):1393-1404. doi: 10.2174/1570159X20666220927112625.	Dupl. 32PM
	ClinicalTrials.gov 11.6.2025 Condition: Bipolar disorder Intervention: Lurasidone Other terms: Pediatric → 8 results	
108	Massachusetts General Hospital. Open-Label Study of Latuda for the Treatment of Mania in Children and Adolescents 6-17 Years Old With Bipolar I, Bipolar II and Bipolar Spectrum Disorder. NCT01932541. 20-August-2013–January 2017. ID 1932541 Withdrawn	No data
109	DelBello MP, Goldman R, Phillips D, Deng L, Cucchiaro J, Loebel A. Efficacy and Safety of Lurasidone in Children and Adolescents With Bipolar I Depression: A Double-Blind, Placebo-Controlled Study. J Am Acad Child Adolesc Psychiatry. 2017;56(12):1015-1025. doi: 10.1016/j.jaac.2017.10.006. Epub 2017 Oct 13.	Dupl. 7PM 4C 5C 5Ps 7Sc
110	Findling RL, Goldman R, Chiu YY, Silva R, Jin F, Pikalov A, Loebel A. Pharmacokinetics and Tolerability of Lurasidone in Children and Adolescents With Psychiatric Disorders. Clin Ther. 2015;37(12):2788-97. doi: 10.1016/j.clinthera.2015.11.001. Epub 2015 Nov 26.	Dupl. 4PM 5Sc
111	Foundation of Hope, North Carolina; University of North Carolina, Chapel Hill. An Open-Label Pilot Study of Lurasidone in Treating Antipsychotic Naive or Quasi-Naive Children and Adolescents. 2012/11/14/–2017/06/14/. ID 1731119, https://clinicaltrials.gov/study/NCT01731119.	No BD
112	DelBello MP, Tocco M, Pikalov A, Deng L, Goldman R. Tolerability, Safety, and Effectiveness of Two Years of Treatment with Lurasidone in Children and Adolescents with Bipolar Depression. J Child Adolesc Psychopharmacol. 2021;31(7):494-503. doi: 10.1089/cap.2021.0040. Epub 2021 Jul 29.	Dupl. 23PM 15C 14Ps 19Sc

113	Merck Sharp & Dohme LLC. An Observational Drug Utilization Study of SYCREST^® (Asenapine) in the United Kingdom 2011/12/21/–2013/04/01/ Organon and Co, Completed 2022/02/04/–2022/02/02/ Observational No Results Submitted https://clinicaltrials.gov/study/NCT01498770; ID 1498770; MK-8274-108.	No data
114	Patient-Centered Outcomes Research Institute; Montana State University; National Alliance on Mental Illness Montana; CGStat LLC; Risk Benefit Statistics LLC; National Alliance on Mental Illness New Mexico; National Alliance on Mental Illness Westside Los Angeles; University of New Mexico. Longitudinal Comparative Effectiveness of Bipolar Disorder Therapies. https://clinicaltrials.gov/study/NCT02893371. Started 2016/08/30/–2016/09// Terminated 2024/03/12/–2024/03/08/	No data
115	The Emmes Company, LLC, Daniel Benjamin, Eunice Kennedy Shriver National Institute of Child Health and Human Development (NICHD). Pharmacokinetics of Understudied Drugs Administered to Children Per Standard of Care (PTN_POPS). https://clinicaltrials.gov/study/NCT01431326. Started 2011/08/17/–2011/11// jkl–Completed 2023/09/06/–2023/08/31/.	No data

Included 10
- Cases 4
- Studies 6

Excluded 105
- Reviews 15
- Adult 7
- Off-target 6
- Opinion 5
- No data 4
- Congress Abstracts 3
- No BD 3
- Lumping 2
- No lurasidone 1
- Duplicates 59

Supplementary Table S2. PRISMA 2020 CheckList

PRISMA

PRISMA 2020 Checklist

Section and Topic	Item #	Checklist item	Location where item is reported
**TITLE**			1
Title	1	Identify the report as a systematic review.	1
**ABSTRACT**			1
Abstract	2	See the PRISMA 2020 for Abstracts checklist.	1
**INTRODUCTION**			1-4
Rationale	3	Describe the rationale for the review in the context of existing knowledge.	4
Objectives	4	Provide an explicit statement of the objective(s) or question(s) the review addresses.	4
**METHODS**			4
Eligibility criteria	5	Specify the inclusion and exclusion criteria for the review and how studies were grouped for the syntheses.	4
Information sources	6	Specify all databases, registers, websites, organisations, reference lists and other sources searched or consulted to identify studies. Specify the date when each source was last searched or consulted.	4
Search strategy	7	Present the full search strategies for all databases, registers and websites, including any filters and limits used.	4
Selection process	8	Specify the methods used to decide whether a study met the inclusion criteria of the review, including how many reviewers screened each record and each report retrieved, whether they worked independently, and if applicable, details of automation tools used in the process.	4
Data collection process	9	Specify the methods used to collect data from reports, including how many reviewers collected data from each report, whether they worked independently, any processes for obtaining or confirming data from study investigators, and if applicable, details of automation tools used in the process.	4
Data items	10a	List and define all outcomes for which data were sought. Specify whether all results that were compatible with each outcome domain in each study were sought (e.g. for all measures, time points, analyses), and if not, the methods used to decide which results to collect.	4
	10b	List and define all other variables for which data were sought (e.g. participant and intervention characteristics, funding sources). Describe any assumptions made about any missing or unclear information.	4
Study risk of bias assessment	11	Specify the methods used to assess risk of bias in the included studies, including details of the tool(s) used, how many reviewers assessed each study and whether they worked independently, and if applicable, details of automation tools used in the process.	N/A
Effect measures	12	Specify for each outcome the effect measure(s) (e.g. risk ratio, mean difference) used in the synthesis or presentation of results.	N/A
Synthesis methods	13a	Describe the processes used to decide which studies were eligible for each synthesis (e.g. tabulating the study intervention characteristics and comparing against the planned groups for each synthesis (item #5)).	4
	13b	Describe any methods required to prepare the data for presentation or synthesis, such as handling of missing summary statistics, or data conversions.	N/A
	13c	Describe any methods used to tabulate or visually display results of individual studies and syntheses.	4
	13d	Describe any methods used to synthesize results and provide a rationale for the choice(s). If meta-analysis was performed, describe the model(s), method(s) to identify the presence and extent of statistical heterogeneity, and software package(s) used.	N/A

Section and Topic	Item #	Checklist item	Location where item is reported
	13e	Describe any methods used to explore possible causes of heterogeneity among study results (e.g. subgroup analysis, meta-regression).	N/A
	13f	Describe any sensitivity analyses conducted to assess robustness of the synthesized results.	N/A
Reporting bias assessment	14	Describe any methods used to assess risk of bias due to missing results in a synthesis (arising from reporting biases).	N/A
Certainty assessment	15	Describe any methods used to assess certainty (or confidence) in the body of evidence for an outcome.	N/A
**RESULTS**			4-8
Study selection	16a	Describe the results of the search and selection process, from the number of records identified in the search to the number of studies included in the review, ideally using a flow diagram.	5
	16b	Cite studies that might appear to meet the inclusion criteria, but which were excluded, and explain why they were excluded.	Suppl.
Study characteristics	17	Cite each included study and present its characteristics.	5-8
Risk of bias in studies	18	Present assessments of risk of bias for each included study.	N/A
Results of individual studies	19	For all outcomes, present, for each study: (a) summary statistics for each group (where appropriate) and (b) an effect estimate and its precision (e.g. confidence/credible interval), ideally using structured tables or plots.	N/A
Results of syntheses	20a	For each synthesis, briefly summarise the characteristics and risk of bias among contributing studies.	N/A
	20b	Present results of all statistical syntheses conducted. If meta-analysis was done, present for each the summary estimate and its precision (e.g. confidence/credible interval) and measures of statistical heterogeneity. If comparing groups, describe the direction of the effect.	N/A
	20c	Present results of all investigations of possible causes of heterogeneity among study results.	N/A
	20d	Present results of all sensitivity analyses conducted to assess the robustness of the synthesized results.	N/A
Reporting biases	21	Present assessments of risk of bias due to missing results (arising from reporting biases) for each synthesis assessed.	N/A
Certainty of evidence	22	Present assessments of certainty (or confidence) in the body of evidence for each outcome assessed.	N/A
**DISCUSSION**			8-11
Discussion	23a	Provide a general interpretation of the results in the context of other evidence.	8-9
	23b	Discuss any limitations of the evidence included in the review.	11
	23c	Discuss any limitations of the review processes used.	11
	23d	Discuss implications of the results for practice, policy, and future research.	11
**OTHER INFORMATION**			
Registration and protocol	24a	Provide registration information for the review, including register name and registration number, or state that the review was not registered.	4
	24b	Indicate where the review protocol can be accessed, or state that a protocol was not prepared.	4
	24c	Describe and explain any amendments to information provided at registration or in the protocol.	N/A
Support	25	Describe sources of financial or non-financial support for the review, and the role of the funders or sponsors in the review.	11
Competing interests	26	Declare any competing interests of review authors.	11
Availability of data, code and other materials	27	Report which of the following are publicly available and where they can be found: template data collection forms; data extracted from included studies; data used for all analyses; analytic code; any other materials used in the review.	N/A

Supplementary Table S3. Quality assessment of included studies.

JBI Critical Appraisal Checklist for Case Reports

Study: Channing et al., 2018 [32]				
Question	Yes	No	Unclear	Not applicable
1. Were patient's demographic characteristics clearly described?	X			
2. Was the patient's history clearly described and presented as a timeline?	X			
3. Was the current clinical condition of the patient on presentation clearly described?	X			
4. Were diagnostic tests or assessment methods and the results clearly described?	X			
5. Was the intervention(s) or treatment procedure(s) clearly described?	X			
6. Was the post-intervention clinical condition clearly described?	X			
7. Were adverse events (harms) or unanticipated events identified and described?	X			
8. Does the case report provide takeaway lessons?	X			
Study: Prieto et al., 2019 [33]				
Question	Yes	No	Unclear	Not applicable
1. Were patient's demographic characteristics clearly described?	X			
2. Was the patient's history clearly described and presented as a timeline?	X			
3. Was the current clinical condition of the patient on presentation clearly described?	X			
4. Were diagnostic tests or assessment methods and the results clearly described?	X			
5. Was the intervention(s) or treatment procedure(s) clearly described?	X			

6. Was the post-intervention clinical condition clearly described?	X			
7. Were adverse events (harms) or unanticipated events identified and described?	X			
8. Does the case report provide takeaway lessons?	X			
Study: Nair et al., 2021 [27]				
Question	Yes	No	Unclear	Not applicable
1. Were patient's demographic characteristics clearly described?	X			
2. Was the patient's history clearly described and presented as a timeline?	X			
3. Was the current clinical condition of the patient on presentation clearly described?	X			
4. Were diagnostic tests or assessment methods and the results clearly described?	X			
5. Was the intervention(s) or treatment procedure(s) clearly described?	X			
6. Was the post-intervention clinical condition clearly described?	X			
7. Were adverse events (harms) or unanticipated events identified and described?	X			
8. Does the case report provide takeaway lessons?	X			
Study: Mole et al., 2022 [34]				
Question	Yes	No	Unclear	Not applicable
1. Were patient's demographic characteristics clearly described?	X			
2. Was the patient's history clearly described and presented as a timeline?	X			
3. Was the current clinical condition of the patient on presentation clearly described?	X			
4. Were diagnostic tests or assessment methods and the results clearly described?	X			
5. Was the intervention(s) or treatment procedure(s) clearly described?	X			
6. Was the post-intervention clinical condition clearly described?	X			
7. Were adverse events (harms) or unanticipated events identified and described?	X			
8. Does the case report provide takeaway lessons?	X			

[29] Moola, S.; Munn, Z.; Tufanaru, C.; Aromataris, E.; Sears, K.; Sfetcu, R.; Currie, M.; Qureshi, R.; Mattis, P.; Lisy, K.; Mu, P.-F. Chapter 7: Systematic reviews of etiology and risk. In: Aromataris, E.; Munn, Z. (Editors). *JBI Manual for Evidence Synthesis*. Joanna Briggs Institute, Adelaide, South Australia, 2020. Available from https://synthesismanual.jbi.global.

[30] Gagnier, J.J.; Kienle, G.; Altman, D.G.; Moher, D.; Sox, H.; Riley, D.; CARE Group. The CARE guidelines: consensus-based clinical case reporting guideline development. *Headache* **2013**, *53*(10), 1541-1547. doi: 10.1111/head.12246.

Cochrane tool for assessing risk of bias in randomised trials (RoB-2)

**Study**	**Confounding**	**Measurement of exposure**	**Participant selection**	**Post-exposure intervention**	**Missing data**	**Measurement of outcome**	**Selection of reported results**	**Overall Risk of Bias**
DelBello et al., 2017 [19]								
Singh et al., 2020 [25]								
DelBello et al., 2021 [26]								
Kadakia et al., 2021 [35]								
Diao et al., 2022 [36]								
Singh et al., 2023 [37]								

Colour code: Red, high risk of bias; Yellow, some concerns; Green, low risk of bias.

[31] Sterne, J.A.C.; Savović, J.; Page, M.J.; Elbers, R.G.; Blencowe, N.S.; Boutron, I.; Cates, C.J.; Cheng, H.-Y.; Corbett, M.S.; Eldridge, S.M.; Hernán, M.A.; Hopewell, S.; Hróbjartsson, A.; Junqueira, D.R.; Jüni, P.; Kirkham, J.J.; Lasserson, T.; Li, T.; McAleenan, A.; Reeves, B.C.; Shepperd, S.; Shrier, I.; Stewart, L.A.; Tilling, K.; White, I.R.; Whiting, P.F.; Higgins, J.P.T. RoB 2: a revised tool for assessing risk of bias in randomised trials. *BMJ* **2019**, *366*, l4898.
